# Human Life History is Shaping Embodied Medicinal Plant Knowledge Across the Lifespan

**DOI:** 10.1007/s12110-026-09527-y

**Published:** 2026-07-08

**Authors:** Jorge Izaquiel Alves de Siqueira, Patrícia Muniz de Medeiros, Ulysses Paulino Albuquerque

**Affiliations:** 1 Laboratório de Ecologia e Evolução de Sistemas Socioecológicos (LEA), Universidade Federal de Pernambuco (UFPE), Recife, Pernambuco Brazil; 2 Laboratório de Ecologia, Conservação e Evolução Biocultural (LECEB), Universidade Federal de Alagoas (UFAL), Rio Largo, Alagoas Brazil

**Keywords:** Disease events, Evolutionary ethnobiology, Gender roles, Human health-seeking behavior, Intracultural variation of local knowledge, Local ecological knowledge, Comportamento humano em busca de saúde, Conhecimento ecológico local, Etnobiologia evolutiva, Eventos de adoecimento, Papéis de gênero, Variação intracultural do conhecimento local

## Abstract

In this study, we draw on theoretical frameworks from Life History Theory, and Embodied Capital Theory to assess the role of human life history in shaping local medical systems (LMSs) across the lifespan. We focus specifically on medicinal plants, as they can enhance individual fitness and contribute to overall health. Our central research question is: *What role does human life history play in the construction of LMSs?* To address this, we conducted an ethnobiological investigation in a small rural mixed-subsistence community in northern Piauí, Northeastern Brazil. We examined four key dimensions: (1) medicinal plant composition and therapeutic targets across the lifespan; (2) embodied medicinal plant knowledge (EMPK) strongly associated with specific life phases; (3) the influence of gender modulated by life history; and (4) medicinal plant knowledge strongly associated with men and women. Our findings indicate that (1) human life history shapes medicinal plant knowledge across the lifespan; (2) certain medicinal plants and therapeutic targets are strongly associated with the reproductive and post-reproductive phases; (3) EMPK between men and women differs only in reproductive phase; and (4) a distinct set of medicinal plants and therapeutic targets is strongly associated with women, but not with men. We argue that human life history structures life-phase-specific patterns of medicinal plant knowledge. These insights provide novel perspectives on how humans construct LMSs and are particularly relevant for understanding the differentiated contributions of men and women to these systems throughout the life course.

**Supplementary Information:**

The online version contains supplementary material available at https://doi.org/10.1007/s12110-026-09527-y.

## Resumo

Nesta pesquisa, exploramos aportes teóricos da Teoria da História de Vida, Teoria do Capital Incorporado e Hipótese da Avó para avaliar o papel da história de vida humana na construção de sistemas médicos locais (SMLs) ao longo da vida. Nossos esforços de pesquisa se concentraram particularmente em plantas medicinais porque elas podem incrementar o *fitness* das pessoas e contribuir para a qualidade da saúde. Dessa forma, nosso esforço central de investigação foi responder: *Qual é o papel da história de vida humana na construção de SMLs?* Para responder a essa pergunta, conduzimos um estudo etnobotânico em uma pequena comunidade rural de subsistência mista no norte do Piauí, Nordeste do Brasil. Focamos em quatro eixos principais: (1) composição de plantas medicinais + alvos terapêuticos ao longo da vida; (2) conhecimento incorporado sobre plantas medicinais (CIPM) fortemente associado a certos estágios de vida; (3) o papel do gênero modulado pela história de vida; e (4) o conhecimento incorporado de plantas medicinais fortemente associado a homens e mulheres. Nossas contribuições indicaram que (1) a história de vida humana molda o conhecimento sobre plantas medicinais ao longo da vida; (2) que há plantas medicinais e alvos terapêuticos fortemente associados aos estágios reprodutivo e pós-reprodutivo; (3) homens e mulheres apresentam CIPM diferenciado apenas no estágio reprodutivo; e (4) que há um conjunto de plantas medicinais e alvos terapêuticos fortemente associados às mulheres e não aos homens. Discutimos que a história de vida humana molda diferentes padrões de conhecimento sobre plantas medicinais apropriados para cada estágio de vida. Esses *insights* fornecem novas perspectivas sobre como nós seres humanos construímos nossos SMLs. Essas descobertas são particularmente relevantes para ampliar nossa compreensão dos papeis que homens e mulheres em diferentes estágios de vida desempenham na construção desses sistemas ao longo da vida.

## Introduction

In this study, we investigate the role of human life history in shaping medicinal plant knowledge within local medical systems (LMSs). Understanding how our species builds, maintains, and enriches its plant knowledge systems has been a central focus of ethnobiological research (Albuquerque et al. 2019a). Within these systems, LMSs are included as strategies to address various health-related challenges (Dunn, 1976; Kleinman, 1978; Albuquerque et al., 2024). Numerous studies have examined the drivers behind the construction of LMSs by human species (see Silva et al., 2011; Melo et al., 2021; Sousa et al., 2023). However, how medicinal plant knowledge develops and differentiates across the human lifespan remains insufficiently understood. A life history-centered approach offers a promising framework for addressing this knowledge gap (see Hawkes et al., 1989; Stearns, 1992; Roff, 1993; Kaplan, 1996; Schniter et al., 2015, 2021; Siqueira et al., 2026).

## LMSs as a Human Strategy to Deal with Disease Events

Different factors have been studied for their influence on LMSs structure, including income, level of schooling, gender (Díaz-Reviriego et al., 2016; Gomes et al., 2024), migration (Kujawaska & Schmeda-Hirschmann, 2022), occupation (Silva et al., 2011), urbanization (Arjona-García et al., 2021), family context (Brito et al., 2019), marital status (Dessie & Amsalu, 2024), age (Melo et al., 2021), taste perception (Brito et al., 2024), perceived efficacy (Sousa et al., 2023; Beyene et al., 2024), interaction with other LMSs and biomedicine (Abreu et al., 2023; Siqueira et al., 2024), human-modified landscapes and their interplay with socioeconomic status (Cantalice et al., 2025), prior disease experience (Santos et al., 2023), and local consensus (Sousa et al., 2023), among others. Despite these efforts, a key knowledge gap remains: understanding how medicinal plant knowledge develops and diversifies throughout the human lifespan.

Within these scenarios, human behavior related to the use, management, and transmission of natural resource knowledge is shaped by multiple factors. In this study, we focused specifically on one of them: human life history (Schniter et al., 2015, 2021).

Exploring the role of life history in LMSs construction requires recognizing that individuals must overcome various challenges to maximize their fitness—including predators, infections, parasites, and diseases (Stearns, 1992; Roff, 1993). Managing disease pressure is a particularly relevant challenge for our species (Fabrega, 1997). In this context, LMSs emerge as adaptive strategies to restore health and mitigate disease-related stressors (Albuquerque et al., 2024). These systems are dynamic and complex, reflecting behavioral patterns and strategies essential for health maintenance (Dunn, 1976; Kleinman, 1978; Albuquerque et al., 2024). They also are considered biocultural entities shaped by the interaction of biological, ecological, and cultural factors (Ferreira-Júnior & Albuquerque, 2018). Within these scenarios, investing time and energy in learning about medicinal plants for disease prevention, alleviation, and treatment may be critical to both survival and reproductive success in human species (Fabrega, 1997).

## Human Life History

How, then, can the distinct characteristics of human life history contribute to broadening our understanding of the construction of LMSs across the lifespan? This question requires us to examine the defining features of each human life phase. A key biological distinction of the human species is its unusual life history trajectory (Gopnik et al., 2017), which includes an extended pre-reproductive phase, a relatively short reproductive period, and a long post-reproductive lifespan (Jones, 2011). These life history traits enable the exploitation of culture as an adaptive advantage (Richerson & Boyd, 2020), including the acquisition and transmission of medicinal plant knowledge within different social-ecological systems.

Individuals at different life phases engage with medicinal plant knowledge differently (Guimbo et al., 2011; Bruschi et al., 2019; Schniter et al., 2015, 2021; Souza et al., 2021; Siqueira et al., 2026), resulting in intracultural variation—i.e., differential knowledge distribution among individuals. This variation is vital for the structure, functionality, sustainability, and evolution of LMSs. Certain skills, competencies and abilities associated with medicinal plant use are acquired early in life, while others are developed later (Shukla & Sinclair, 2009), leading to the structuring of diverse knowledge profiles across the human lifespan (Schniter et al., 2015, 2021; Pretelli et al., 2022).

In this way, children and adolescents in pre-reproductive phase are cared for by adults and heavily influenced by their knowledge and experiences (Lee, 2013). Key adaptive information is acquired during this formative phase (Barrett et al., 2016; Gallois et al., 2018). This period, marked by intense physical, cognitive, and social development, is also when important cultural knowledge—such as ecological and medicinal plant knowledge—is internalized (Kaplan et al., 2003; Gallois & Reyes-García, 2018). Children may possess distinct knowledge sets not retained into adulthood (Bruschi et al., 2019; Sousa et al., 2022), and they also can hold extensive knowledge of medicinal plants (Gallois et al., 2024). Human childhood, shaped by natural selection, evolved as a phase of exploration, flexibility, and intense learning (Richerson & Boyd, 2020). In addition to intensive learning, at this phase children can also teach others and produce different and relevant innovations in local knowledge systems (Lew-Levy & Amir, 2024). Individuals in this phase have no offspring and thus face different evolutionary pressures than adults (Stearns, 1992; Roff, 1993; Kaplan, 1996; Bogin, 1997).

In the reproductive phase, knowledge regarding natural resources continues to develop, especially in relation to food and toxic plants. Adults acquire knowledge tied directly to subsistence practices and the care of dependent offspring (Schniter et al., 2021). New responsibilities or demands emerge at this life phase—including caregiving—demand broader and more specialized knowledge of medicinal resources, particularly for children’s healthcare. Thus, adults tend to exhibit a broader knowledge associated with natural resources than individuals in the pre-reproductive phase (Schniter et al., 2021). In the post-reproductive phase, individuals typically reduce their participation in physically demanding tasks. Age-related cognitive and physical declines may affect the recall and use of information relevant for human survive (Craik & Rose, 2012), such as the medicinal plant knowledge for dealing disease events. Although this knowledge reaches a peak during adulthood, it may decline or become less accessible in older age (Koster et al., 2020). Nevertheless, this later human life phase remains critical for knowledge retention and transmission.

## Life History Theories and Evolutionary Ethnobiology

Life history theory offers valuable insights into how humans interact with natural resources (e.g., Schniter et al., 2015, 2021; Pretelli et al., 2022), providing a theoretical foundation for predicting how LMSs are constructed across the human lifespan (Stearns, 1992; Roff, 1993; Hawkes et al., 1989; Kaplan, 1996). Understanding the development of LMSs throughout the life course is critical for identifying how knowledge of medicinal plants emerges, evolves, and differentiates in our species.

Three complementary theoretical frameworks are particularly useful for explaining how LMSs are structured over time: Life History Theory-LHT (Stearns, 1992; Roff, 1993), Embodied Capital Theory-ECT (Kaplan, 1996), and the Grandmother Hypothesis-GH (Hawkes et al., 1989). These theoretical frameworks address ecological and evolutionary dynamics across different life phases, suggesting that individuals within the same phase share more strategies and behaviors with each other than with those at different phases (Hawkes et al., 1989; Stearns, 1992; Roff, 1993). We propose that these life phase-specific strategies are reflected in the ways humans construct LMSs, reflecting on the structuring of different patterns of knowledge about medicinal plants across the human lifespan.

Classical LHT seeks to explain how natural selection and other evolutionary forces shape organisms to maximize survival and reproduction under varying ecological conditions. It addresses variation in traits such as birth size, growth rate, age and size at maturity, clutch size, reproductive investment, mortality rates, and lifespan (Stearns, 1992; Roff, 1993). At its core, LHT focuses on trade-offs between survival and reproduction: organisms cannot allocate unlimited energy to both simultaneously (Law, 1979). This necessitates strategic decisions about how to invest limited energy resources (Sear, 2020). These decisions may include learning, transmitting, and practicing medicinal plant knowledge as part of LMSs. As individuals age, they develop adaptive strategies to optimize reproduction and ensure offspring quality (Kaplan, 1996), including strategies for managing disease events (Fabrega, 1997). Thus, the construction of LMSs may reflect underlying life history strategies evolved to address these challenges.

These frameworks also help us understand the life history traits that distinguish humans from other species. From the perspective of ECT, biological fitness depends on how individuals and parents invest in the development and maintenance of physical capital (e.g., muscles, digestive organs, brain), and functional capital^1^ (e.g., immune function, motor skills, knowledge) (Kaplan, 1996; Kaplan & Bock, 2001; Kaplan et al., 2003). This theory predicts increased productivity and parental investment during the reproductive phase and first years in post-reproductive period, driven by the caloric and experiential needs of offspring, with reduced investment once children become more independent—especially by the end of the pre-reproductive phase (Kaplan, 1996; Schniter et al., 2021).

Furthermore, ECT suggests that certain ages are more critical than others in terms of energy investment in physical and cognitive capital, both for the self and for offspring (Kaplan et al., 2003). A plateau of maximum productivity is expected, followed by a decline in investment later in life (Kaplan & Bock, 2001). This aligns with ethnobotanical evidence showing that knowledge about plants peaks at certain ages (Albuquerque et al., 2011; Abreu et al., 2015). ECT predicts that embodied knowledge—including medicinal plant knowledge—develops and diversifies throughout the life course as individuals grow, reproduce, and invest in child-rearing and later-life support (Kaplan, 1996; Kaplan et al., 2003; Schniter et al., 2021). As environmental and familial demands increase, so too does the need for specialized knowledge. This suggests that human life history not only supports the acquisition of relevant knowledge for survive (such as medicinal plant knowledge), but also structures it in age-appropriate profiles (Del Giudice et al., 2015).

Together, LHT and ECT illuminate how variations in life phases and embodied capital investment influence human–nature interactions. The GH adds another dimension, providing a framework for understanding the evolutionary importance of the post-reproductive period in the construction of LMSs. GH emphasizes that reproductive success in humans is supported by post-reproductive individuals, particularly grandmothers, whose contributions may have enabled earlier weaning and shorter birth intervals (Hawkes et al., 1989; Kaplan, 1997; Hawkes, 2020). Longevity after menopause distinguishes humans from our closest living evolutionary cousins, and may have evolved in our lineage when economic productivity and support from grandmothers allowed mothers to wean earlier and shorten births between children (Hawkes et al., 1989).

This life phase is particularly relevant for transmitting accumulated knowledge and skills—especially those requiring expertise but not physical strength (Schniter et al., 2023). According to GH (Hawkes et al., 1989; Hawkes, 2003), grandmothers are key contributors to socioecological systems, sharing practical knowledge and cultural capital. ECT complements this by suggesting that both men and women in the post-reproductive phase can contribute meaningfully to family fitness by transmitting the knowledge gained through a lifetime of experience. This knowledge, particularly about medicinal plants, can serve as a crucial asset in navigating environmental stressors and maintaining community health.

Within the ethnobiological context, particularly in LMS research, these theories underscore the role of older adults, both male and female, as vital contributors to local healthcare systems. Their knowledge reflects gendered life experiences and may lead to differences in the composition of plants known by each gender. Therefore, when individuals enter the post-reproductive phase, they may contribute uniquely to the construction of local knowledge systems (Schniter et al., 2023).

## Knowledge Strongly Associated Across the Lifespan, and the Role of Gender Modulated by Life History

Knowledge differentiation can result in distinct bodies of knowledge that are exclusive to specific groups. While local ecological knowledge may continue to develop into adulthood, children may retain a unique set of knowledge that is not maintained later in life (Gallois et al., 2017). Accordingly, the composition of knowledge can differ between children and adults (Bruschi et al., 2019; Sousa et al., 2022). Indeed, children can know as much as adults about plants (Gallois et al., 2024). Children, adolescents, and the elderly recognize natural resources differently; items recognized by one group may not be recognized by another (Schniter et al., 2021), reflecting the life-phase structuring of ecological knowledge. Although adults may hold broader knowledge than younger individuals, some knowledge, such as awareness of certain plants or diseases more prevalent in older age, may be specific to life phase (Silva et al., 2011; Souza et al., 2021). For instance, a set of knowledge of medicinal plants considered important by adults may not hold the same relevance for children, leading to differences in the composition of known medicinal plants (Sousa et al., 2022). Ethnobotanical literature often uses the term ‘exclusive’ to describe species known only to certain groups. In this study, however, we refer to species and therapeutic targets as being ‘strongly associated^2^’ with specific life phases.

Another important aspect for life history theories and LMS research is the role of gender as modulated by life history. In many societies, particularly those organized along more patriarchal lines, women play a key role in constructing LMSs, particularly due to their central involvement in family healthcare (Silva et al., 2011). The reproductive and post-reproductive life phases are crucial in the human life history and reflect a suite of traits unique to our species. Analyzing these characteristics offers insights into how gendered roles evolve across life phases and how human life history shapes the interaction between people and plants. Gender has long been recognized as a significant factor in differentiating plant knowledge between men and women (Silva et al., 2011; Almeida et al., 2012; Díaz-Reviriego et al., 2016; Costa et al., 2021), and influences the extent of knowledge sharing in socio-ecological systems (Costa et al., 2021). On a global scale there are no differences in the knowledge of men and women, but on a local scale different results are recorded (Torres-Avilez et al., 2016) and both genders fulfill different roles in the construction of LMSs (see Tadesse et al., 2025).

Differences in ecological knowledge between boys and girls begin in childhood, typically between ages 7 and 12 (Pretelli et al., 2022), and become more structured in adulthood depending on occupational niches and gender roles (Schniter et al., 2021). Although gender is known in shaping LMSs, we still lack a clear understanding of how it is modulated by human life history, particularly in relation to EMPK.

As presented previously, during the reproductive and post-reproductive phases, the dynamics of embodied capital shift significantly compared to the pre-reproductive period. These shifts are often gendered, especially in terms of parental investment (Kaplan, 1996). After the first reproductive event, parents invest not only in their own embodied capital but also in that of their offspring. This investment varies by gender. Research suggests that boys and girls raised by mothers show similar rates of knowledge acquisition, whereas children raised by fathers often exhibit gendered differences—boys tending to gain broader knowledge than girls—indicating a gender bias in paternal investment (Pretelli et al., 2022). These patterns suggest that mothers invest more heavily in their children’s local ecological knowledge, regardless of the child’s gender.

As a result, women—particularly mothers—may develop broader and more specialized knowledge to manage disease events, potentially reflected in the diversity and richness of medicinal plant knowledge compared to men (Silva et al., 2011). Gendered roles thus appear essential to understanding the construction of LMSs across the lifespan (Torres-Avilez et al., 2024). From an early age, girls tend to mention a broader number of medicinal plants than boys (Quinlan et al., 2016), and this gender differentiation regarding ecological knowledge becomes more established during the reproductive phase (Schniter et al., 2021). We therefore hypothesize that the influence of life history on gendered knowledge becomes more pronounced during the reproductive and post-reproductive phases, when demands imposed by disease events are greatest.

Women’s significant role in socio-ecological systems is evident in the health outcomes of their children. Although both men and women contribute to LMSs, in many societies women often play a more active role in household healthcare; this is a culturally contingent pattern rather than a global one. Consequently, parental plant knowledge—especially that of mothers—can positively affect children’s health and well-being (McDade et al., 2007). Maternal knowledge about medicinal plants is associated with lower rates of infection (Miller, 2011; Tanner et al., 2011), and women often possess specialized knowledge for managing children’s diseases (Towns et al., 2014). Their contribution is amplified when they are engaged in nature-related occupations, such as agriculture (Flores-Silva et al., 2021). Moreover, women’s knowledge helps confront environmental challenges, including malnutrition and related diseases (Vissoh et al., 2024). These findings suggest that women may experience significant increases in embodied capital associated with medicinal plant knowledge, which may manifest in differentiated knowledge compositions compared to men after entering the reproductive period.

This scenario becomes more complex when considering the cooperative nature of childrearing. Mothers often receive support from others, challenging the idea of a strictly nuclear family (Sear, 2021). Flexible childcare may have been a key driver in the evolution of human life history (Page et al., 2021). Although humans engage in cooperative breeding and allomaternal care (Sear, 2021), women remain central to LMSs due to their prominent role in family healthcare (Teklehaymanot, 2009; Almeida et al., 2012). When illness occurs, women are generally the first to initiate health-seeking behaviors, thereby shaping a distinct body of EMPK.

Furthermore, men and women may possess knowledge of species that are either exclusive to their gender or shared across genders (Merétika et al., 2010; Albuquerque et al., 2011; Melo et al., 2021). The same pattern holds for therapeutic targets: men and women may experience and treat different health issues with different plant-based remedies (Albuquerque et al., 2011; Souza et al., 2021). Although these have often been labeled as ‘exclusive’ in the literature, we prefer the term ‘strongly associated’ for the same reasons explained above.

Taken together, these findings support the idea that human life history shapes how individuals interact with natural resources. To our knowledge, this is the first study to explicitly investigate the relationship between life history and EMPK across the human lifespan. Previous research has examined the ontogeny of various traditional practices, such as food processing, artisanal production, childrearing (Schniter et al., 2015), foraging practices (Koster et al., 2020), local knowledge regarding of edible and dangerous species (Schniter et al., 2021), and general ecological learning (Lew-Levy et al., 2021; Pretelli et al., 2022).

The gendered patterns we describe in this paper reflect social roles and divisions of labor documented in specific cultural contexts; we invoke evolutionary frameworks not to treat these roles as biologically fixed or universal, but to analyze how life history pressures interact with culturally embedded arrangements to produce the patterns we observe across the lifespan.

## Research Questions, Hypotheses and Predictions

We aim to understand the role of human life history in shaping LMSs. To this end, we evaluate four central research questions and their corresponding hypotheses and predictions:

Question 1: What role does human life history play in the construction of LMSs across the lifespan?

Hypothesis 1 (H1): Human life history shapes medicinal plant knowledge.

Prediction 1 (P1): At each life phase, individuals will exhibit a distinct and well-defined composition of medicinal plants and therapeutic targets. Specifically, the response variable—the combined composition of cited medicinal plants and their therapeutic targets—will vary across the predictor variable (life phase), with compositional similarity being greater within than between phases. Thus, life phase is a key factor in structuring plant knowledge within LMSs.

Question 2: How does life history influence the structuring of medicinal plant knowledge strongly associated with specific life phases?

Hypothesis 2 (H2): Human life history modulates the structuring of plant knowledge strongly associated with specific phases. Whereas H1 tests whether the overall composition of medicinal plant knowledge differs among life phases, H2 goes further by testing whether particular medicinal plants and therapeutic targets can be identified as indicators that are specifically and faithfully associated with a given life phase.

Prediction 2 (P2): Adults in reproductive and post-reproductive phases will show a set of specific medicinal plants strongly associated with these phases, unlike pre-reproductive individuals.

Prediction 3 (P3): Likewise, adults in reproductive and post-reproductive phases will exhibit a set of therapeutic targets strongly associated with these phases, whereas pre-reproductive individuals will not.

Question 3: What is the role of gender, as modulated by life history, in the construction of LMSs?

Hypothesis 3 (H3): Life history modulates the role of gender as a predictor for the development and differentiation of medicinal plant knowledge.

Predictions 4 and 5 (P4, P5): In reproductive and post-reproductive phases, women^3^ will display a broader and more structured composition of medicinal plants (P4), and therapeutic targets (P5) compared to men.

Question 4: How does human life history influence the structuring a set of EMPK associated with genders after first reproduction event or family formation?

Hypothesis 4 (H4): There is a set of medicinal plants and therapeutic targets strongly associated with men and women in reproductive and post-reproductive phases in LMSs.

Predictions 6 and 7 (P6, P7): Thus, we expect to find a set of medicinal plants (P6), and therapeutic targets (P7) strongly associated with men and another set strongly associated with women within LMSs in reproductive and post-reproductive life phases.

## Study Area

**Fig. 1:**
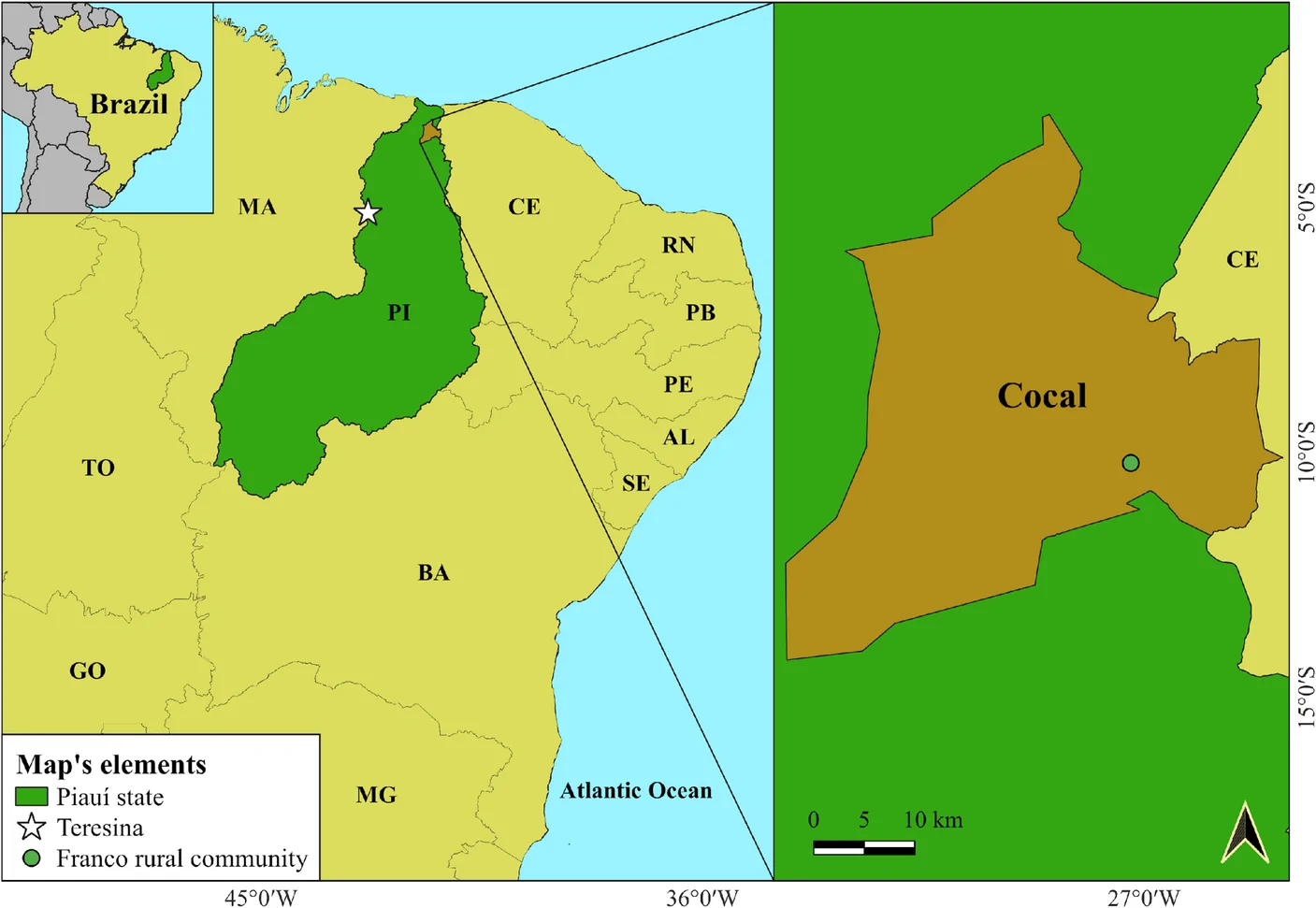
Location map of the Franco rural community, Cocal, Piauí, NE Brazil. Data from IBGE (2022)

Fieldwork was conducted in the Franco rural community (03°29’48.8"S, 41°26’19.5"W), located in the municipality of Cocal, northern Piauí, approximately 268 km from the state capital, Teresina, Northeastern Brazil (Fig. 1). Cocal has a population of 28.212 inhabitants and a demographic density of 21.8 inhabitants/km² (IBGE, 2022). The study area is situated within the boundaries of the *Serra da Ibiapaba* Environmental Protection Area (EPA) (Brasil, 1996). The Franco rural community consists of 103 residents distributed across 30 family units.

The local climate features two well-defined seasons: (1) a rainy summer, and (2) a dry winter. It is classified as hot tropical, with average temperatures ranging between 25 °C and 35 °C, and annual rainfall averaging approximately 900 mm (Medeiros, 2004). The predominant vegetation type is *Carrasco*, with notable representation from botanical families such as Fabaceae, Euphorbiaceae, Bignoniaceae, and Asteraceae (Chaves, 2005).

**Fig. 2:**
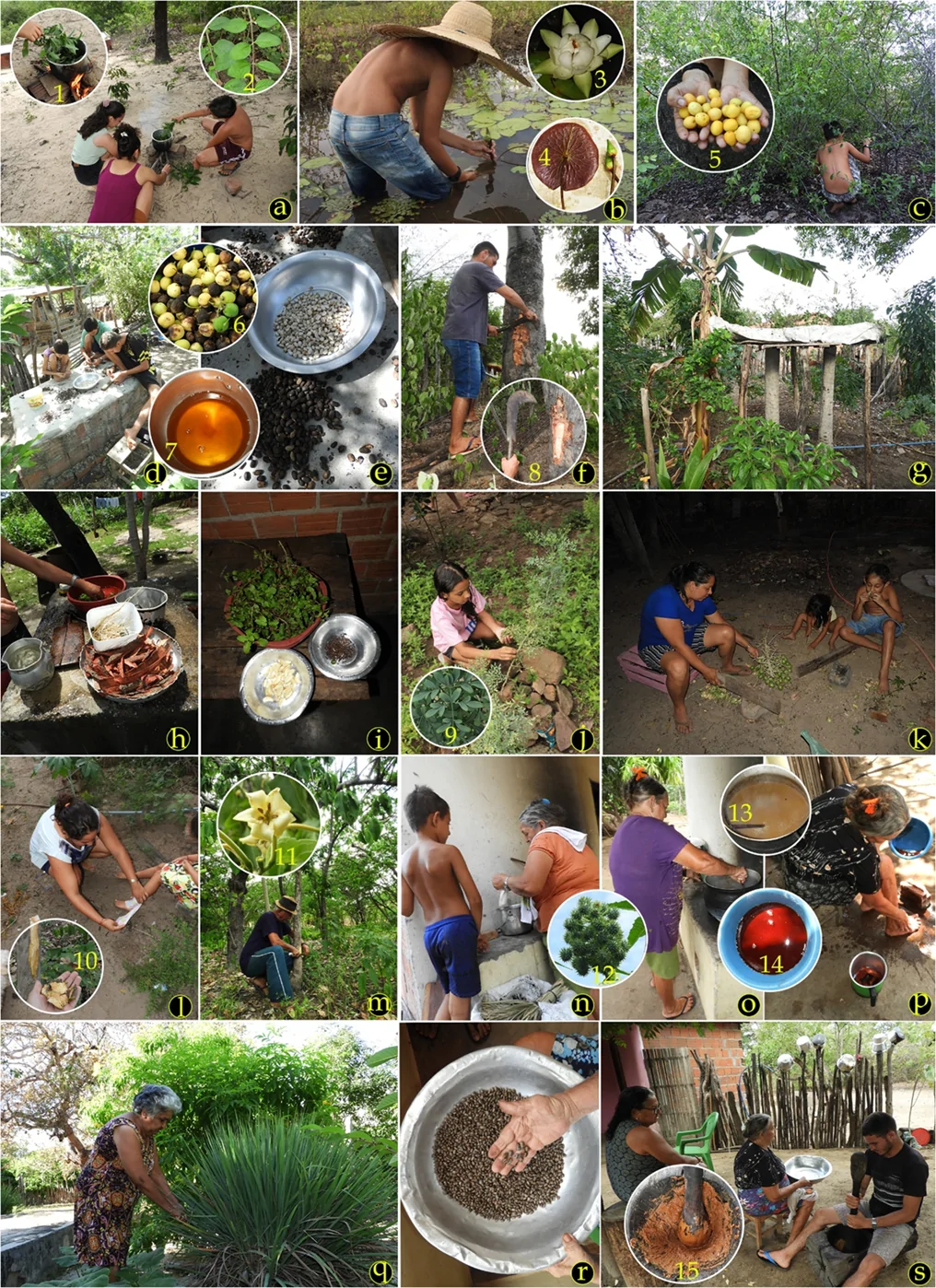
Some details of the use of medicinal plants throughout life in the Franco rural community: (**a**). children playing (1) at making a medicine with *Lippia alba* (Mill.) N.E.Br. ex Britton & P.Wilson branches (2); (**b**). child collecting *Nymphaea amazonum* Mart. & Zucc. leaves (4) in a river; flower details of this species; (**c**). child collecting *Ximenia americana* L. fruits (5) in the forest, an important food and medicinal species; (**d**). children/teenagers removing the cotyledons from *Jatropha curcas* L. seeds for oil (7) extraction; fruits of this species (6); (**e**). details of the cotyledons and dried fruits of *J. curcas*; (**f**). Young adult still in pre-reproductive phase harvesting bark from the stem of *Hymenaea courbaril* L., a practice that requires strength and skill; sickle (8) used to remove bark; (**g**). Hanging garden, where people grow many medicinal plants, especially for children’s healthcare; (**h**-**i**). multiple local species collected to prepare medicinal plant complexes; (**j**). girl collecting *Ruta graveolens* L. leaves (9) in the home garden; (**k**). Mother with her two children breaking the fruits of *Astrocaryum vulgare* Mart., an important food and medicinal species; (**l**). Mother tying *Tocoyena formosa* (Cham. & Schltdl.) K.Schum. stem bark (10) to her daughter’s ankle to treat luxation; (**m**). Elderly person in post-reproductive phase collecting bark from the stem of *T. formosa*; flower (10) details of this species; (**n**). Grandchild observing his grandmother filtering *Ricinus communis* L. oil; fruits (11) details of this species; (**o**). Elderly woman in post-reproductive phase extracting (12) oil from *R. communis;* (**p**). Elderly woman in post-reproductive phase carrying wound with infusion (13) of *X. americana* bark; (**q**). Elderly woman in post-reproductive phase collecting *Cymbopogon citratus* (DC.) Stapf leaves in home garden; (**r**). Details of the seeds of *R. communis*, a species most important to the studied community; (**s**). Young adult in pre-reproductive phase helping two elderly females in macerating the cotyledons of *J. curcas*, which requires a lot of physical effort

Residents have access to a variety of phytophysiognomies and landscapes with differing degrees of anthropogenic alteration, from which they obtain medicinal plants. These environments include home gardens, roadsides, nearby and distant forested areas, public markets and fairs, and even aquatic habitats. Figure 2 illustrates different practices involving the use of medicinal plants by individuals at different life phases. The presence of two distinct seasons—a dry winter and a rainy summer—suggests that certain species may be seasonally available, with some becoming scarce or entirely absent during specific periods of the year.

In May 2009, the collapse of the *Algodões I* Dam caused widespread destruction throughout the community, resulting in significant economic, social, cultural, and ecological impacts (Siqueira et al., 2019). The Franco rural community experienced both a reduction in household numbers and a contraction of their territorial range.

The Franco community has its origins in rural traditions, with livelihoods primarily based on agriculture and animal husbandry. Many male residents also engage in hunting and fishing as supplementary sources of food and leisure. While women do not typically hunt, one young woman in the reproductive phase was observed fishing during the data collection period, also as a leisure activity. Community members cultivate a variety of plant species in their home gardens, primarily for medicinal and food purposes. The community relies heavily on plants, both cultivated in home gardens and harvested from surrounding natural vegetation, and anthropized areas (Siqueira et al. 2020a, b).

**Fig. 3:**
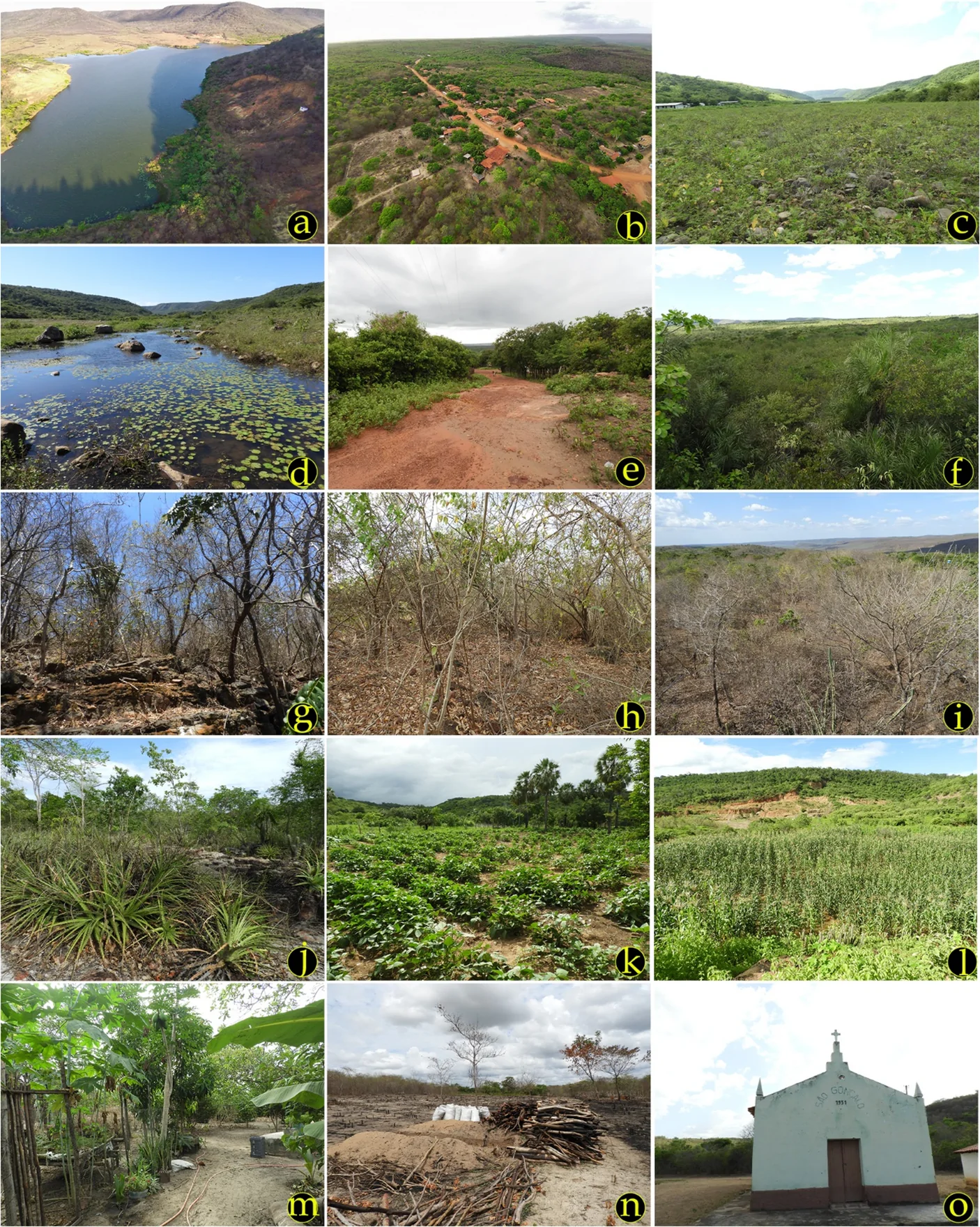
Landscapes and details of the Franco rural community, Cocal-PI: (**a**). view of the *Algodões* I Dam; (**b**). aerial view of an area of the community studied; (**c**). area of the community that was destroyed by the dam collapse, where many families lived; (**d**). Piranji river that runs through the community; (**e**). road that crosses the community; (**f**). secondary forest in the rainy summer; (**g**-**i**). details of the forest in dry winter; (**j**). vegetation that grows in areas of Lajeiros (stony environment), where there are many species of medicinal importance; (**k**-**l**). small areas designated for agriculture, the main local subsistence activity; (**m**). view of a home garden, a space of great importance for livelihood, where people cultivate many species, especially as food and medicinal resource; (**n**). local coal production; (**o**). Catholic Church in the community

There are no schools or primary health care units in the community. However, it does have a Catholic Church and is served by two community health agents. The area lacks basic sanitation and water treatment systems, but electricity is available, and residents have broad access to the internet. Water sources include tubular wells, waterholes, the *Algodões I* Dam, and cisterns. Figure 3 provides visual details of the community landscapes.

Given these characteristics, we selected the Franco rural community as an ideal setting for analyzing the role of human life history in structuring LMSs. This decision was based on several factors: (1) the community’s longstanding dependence on, and deep knowledge of medicinal plants (see Siqueira et al. 2017, 2020a, b); (2) its demographic diversity, including children, adolescents, young adults, adults, and older adults; and (3) logistical advantages, such as ease of access and the first author’s prior field experience in the area, which facilitated rapport-building and enhanced the quality of observational and ethnobotanical data collection.

## Methods

### Ethical and Legal Procedures

The research proposal was submitted for ethical review and approved by the Research Ethics Committee (REC) of the Federal University of Pernambuco under protocol number 5.584.381 (Brasil, 2012, 2016). Given that the study area is located within the *Serra da Ibiapaba* Environmental Protection Area (EPA), authorization for plant collection was obtained from the Authorization and Information System on Biodiversity (SISBIO) under license number 83,490/1 (ICMBio, 2022).

Informational meetings were held with residents to explain the research objectives, procedures, potential benefits, and risks. After reading, discussing, and clarifying the Free and Informed Consent Form (FICF), each resident who voluntarily agreed to participate was invited to sign the document (ISE, 2006). For participants under the age of 18, an Assent Form (AF) was applied, signed by both the minors and their parents or legal guardians.

The study was also registered with the National System for the Management of Genetic Heritage and Associated Traditional Knowledge (SISGEN) under registration number A1FE7F9 (Brasil, 2015). For photographic records in which participants appear, we obtained consent through a separate Image Use Authorization Form (IUAF). These images, which depict interactions involving the use of medicinal plants in the Franco rural community were collected strictly for scientific, non-commercial purposes.

## Participants Selection

All individuals aged five years and older who expressed interest in participating were invited to participate in the study. Our goal was to include as many residents as possible to characterize the investigation as a community census (Albuquerque et al., 2014). The choice to include children from age five was based on the practical difficulty of interviewing younger children and the understanding that cognitive faculties are well developed from this age onward (Suddendorf & Corballis, 1997).

Five children under the age of five were excluded based on the inclusion criteria. Additionally, one resident over 80 years old who was undergoing cancer treatment was not invited to participate. The investigation was organized into three stages, differing in the number of participants. A total of 97 individuals participated in stages I and II of the study (42 women and 55 men). In stage III, 95 individuals participated (42 women and 53 men); two adult male participants in the reproductive phase died during the data collection period. After data validation and consistency checks, the final sample used for hypothesis testing consisted of 90 participants: 41 individuals in the pre-reproductive phase, 21 in the reproductive phase, and 28 in the post-reproductive phase.

### Data Collection

Fieldwork was conducted between October 2022 and September 2023 and was divided into three stages. The data gathered during these stages were used to test the four hypotheses presented in this study. Our data collection strategy initially focused on the structure of EMPK within LMSs, specifically regarding the composition of medicinal plant species and therapeutic targets.

Following the framework proposed by Albuquerque et al. (2019a), we considered three main structuring components of LMSs: (1) the natural resources used (i.e., medicinal plants, assessed by both number and species composition, and their intracultural distribution), (2) individuals and their life history profiles (characteristics defining each life phase), and (3) therapeutic targets (the ailments treated with medicinal plants, analyzed for their distribution and cultural variation across life phases).

In all stages of fieldwork, we employed participant observation to enhance data quality and deepen our understanding of the phenomenon under investigation (Bernard, 2006). Stage I – Characterization and Description of Individual Life History Profiles. In this initial stage, we conducted semi-structured interviews (Albuquerque et al. 2019b), and applied direct observation techniques (Bernard, 2006). These methods were used to collect individual data allowing us to classify participants into life phases. The information gathered included date of birth, gender, marital status, date of marriage or family formation, onset of menopause (for women), expectations of future reproduction, and dependency status regarding offspring or caregivers.

For the purposes of this research, we defined life phases as follows (Table 1):

**Table 1:** Data and descriptions for allocating individuals into life periods, based on theories or approaches focused on life history

Cases	Life phase	References	Additional descriptions
Child, adolescent, young adult who has never had children or formed a family	Pre-reproductive	Stearns (1992), Roff (1993), Jones (2011), Schniter et al. (2015, 2021)	This category includes children, adolescents or young adults who do not yet have children or have formed a family
The individual has children or formed a family	Reproductive	Stearns (1992), Roff (1993), Jones (2011), Schniter et al. (2015, 2021)	This category includes all individuals who formed a family or had children
Women of postmenopausal age	Post-reproductive	Stearns (1992), Hawkes et al. (1989, 2003), Jones (2011 ), Schniter et al. (2015, 2021)	Women who may have already started a family or have children (or not) and who have already entered the menopause period
Men whose partner has already gone through menopause, regardless of age	Post-reproductive	Stearns (1992), Jones (2011), Levitis and Lackey (2011), Schniter et al. (2015, 2021)	Men who form a couple with a woman who has entered menopause and no longer have any expectations of having children

- Pre-reproductive period: from birth (with inclusion beginning at age five due to study design constraints) until the first reproductive event or the formation of a family unit.
- Reproductive period: from the first reproductive event or formation of a family (civil, religious, or informal union) until the cessation of reproduction. For women, this period ends at menopause. Individuals who had not reproduced but had formed family units were also included here, as family formation is assumed to increase social responsibilities and resource demands.
- Post-reproductive period: this category included individuals who had completed the reproductive period—defined for women as post-menopause—and who no longer expected to have children. For men, this classification followed the reproductive status of their female partners, as their reproductive trajectories differ biologically (see Levitis & Lackey, 2011).

Based on the criteria outlined above, we excluded five individuals from our analyses due to ambiguous characteristics and life experiences that prevented clear classification into any of the defined life phases. Consequently, our analyses, results, and discussions are based on data from the remaining 90 participants. Table 2 provides a descriptive summary of the number of individuals within each life phase. Additional information regarding the excluded cases, including the specific criteria for exclusion, is provided in the Supplementary Materials.

**Table 2:** Data and descriptions for allocating the 90 individuals into life periods, based on theories or approaches centered on life history

	Life phase		
	Pre-reproductive	Reproductive	Post-reproductive
Man	29	11	12
Woman	12	10	16
Total	41	21	28
Age range	7–28	22–58	42–88

We used conceptual frameworks from LHT (Stearns, 1992; Roff, 1993), ECT (Kaplan, 1996), and the GH (Hawkes et al., 1989; Hawkes, 2003, 2020) to clearly define the characteristics and dynamics of each life phase—our primary explanatory variable—and to categorize participants accordingly.

#### Stage II – Gathering Data on Memory Associated with the Use of Medicinal Plants

To assess recall-based knowledge (memory) associated with medicinal plants, we employed the free listing technique (Albuquerque et al. 2019b). Participants were individually invited to list medicinal plants they knew or had heard of. To standardize data collection across individuals, we used the following question: (1) *Could you tell me which plants are used as medicine that you know or have heard of?* We clarified that participants could mention any plant-derived product, including those from home gardens, roadsides, forests (near or distant), as well as plants acquired at public fairs or markets. This instruction aimed to minimize bias in which participants might otherwise limit their responses to plants from only certain environments. After participants completed their lists, we used semi-structured interviews (Albuquerque et al. 2019b) to document the therapeutic targets (TTs), and associated symptoms for each plant cited. We asked the following additional questions: *(2) What type of health problem is this plant used for? (3) What symptoms does a person experience when they have this health problem?*

These questions were essential for the standardization of TTs, as multiple symptoms (e.g., phlegm, fever, cough) can correspond to a single TT (e.g., flu), and similar symptoms may sometimes represent distinct TTs. This level of detail was necessary to avoid under or overestimating the models used in subsequent analyses.

#### Stage III – Capturing Data on Knowledge Associated with Medicinal Plants

To assess participants’ knowledge of medicinal plants beyond recall, we used a photographic checklist technique that relied on visual stimuli. This method offers a broader cognitive challenge than free listing and allows for more comprehensive data collection. The checklist was based on the species cited during Stage II, verified through botanical identification (see the Botanical Material section). Plant photographs were taken using a semi-professional camera.

The photographic checklist included 167 species, each represented by multiple images (morphological growth forms, leaves, flowers, fruits, seeds, bark, etc.). These were organized alphabetically by family and genus, following the taxonomy described in the Botanical Material section, and compiled into a PowerPoint^®^ presentation. To minimize order effects and recognition bias, we randomized the image sequence (1–167) using R software version 4.1.0 (R Core Team, 2021). The randomization followed two constraints:

1. An even-numbered image was never followed by an odd-numbered one (or vice versa), to avoid consecutively showing species from the same genus.
2. No five consecutive images could include all five species from a single genus (e.g., *Turnera* spp.), avoiding genus clustering.

After randomization, the photo sequence was rearranged accordingly. Each participant viewed the images on a portable computer and was asked to provide information about each species. Initial questions included: *Do you recognize this plant?* If the plant was recognized, we asked for vernacular name(s), whether the plant was used medicinally, if medicinal, the therapeutic targets (and symptoms).

Participants demonstrated varying levels of familiarity with the plants presented in this stage, which we categorized as follows:

1. Recognizes the plant, knows its name, and identifies medicinal uses;
2. Recognizes the plant, does not know its name, but identifies medicinal uses;
3. Recognizes the plant, cannot recall the name, but identifies medicinal uses;
4. Recognizes the plant, knows its name, but reports no medicinal use;
5. Recognizes the plant, does not know the name, and reports no medicinal use;
6. Recognizes the plant, cannot recall the name, and reports no medicinal use;
7. Does not recognize the plant.

Only responses in categories 1–3 were included in our analyses. A plant was considered ‘recognized’ if the participant provided a correctly vernacular name or accurately described habitat, odor, texture, or morphological features—even if they could not elaborate on health-related uses shown in the photographs. All responses were recorded in structured forms during the interview process.

To ensure high data quality during fieldwork—particularly in stages II and III—we adopted a series of methodological precautions. Firstly, we avoided conducting interviews in situations where many people were present at the same location, as this could lead to participant discomfort, interruptions during the free listing process, or biased responses. Whenever possible, participants were invited to move to a quieter location where others could not overhear or interfere with their answers. The choice of location was left entirely to the participant, allowing them to choose the environment in which they felt most comfortable.

To prevent premature information diffusion, we asked participants not to disclose to others the names of the plants they had listed or recognized during the photographic checklist. These details were treated as confidential to reduce potential influence on the responses of other participants. When interference by third parties was detected, we checked and annotated such occurrences to assess their potential impact on data quality. Additionally, we explicitly requested that third parties refrain from interfering with interviews.

In cases where it was not possible to prevent individuals from overhearing interviews—particularly among members of the same household or close neighbors—we scheduled data collection at different times, ensuring that each participant responded independently. We also refrained from collecting data when participants were visibly busy or in a hurry, as this could compromise the completeness of the free listing or the attention required during the photographic checklist. Finally, we avoided applying any data collection technique when participants showed signs of fatigue, illness, or general discomfort.

These protocols were essential not only for minimizing response bias and ensuring data consistency but also for maintaining ethical standards and respecting the autonomy and well-being of all participants throughout the research process.

### Botanical Material

The plant species mentioned by participants were collected and processed following the protocol described by Mori et al. (1989). To ensure accurate botanical identification, we adopted a two-tiered validation approach. At the first level, local validation was conducted using the guided tour technique (Bernard, 2006), in which participants were invited to walk with the researchers to the places where the plants naturally occurred. In cases where participants were unable to accompany the team due to physical limitations or time constraints, they were asked to describe plant characteristics and the approximate location where the species could be found. Researchers then collected and photographed the specimens, and fresh samples and images were subsequently validated by the participants. For species that were listed but did not occur locally (e.g., wild, spontaneous, or purchased at public markets), identification was based on photographic material. These cases typically referred to plants participants had encountered in other locations where they had previously lived. With the assistance of family members, plants and products not found in the study area or in the nearby urban center of Cocal were photographed exclusively for scientific identification purposes, and were therefore excluded from the photographic checklist used in data collection.

At the second level, scientific identification was conducted through a combination of resources, including consultations with taxonomic literature, visits to the *Delta do Parnaíba* Herbarium (HDELTA, *Universidade Federal do Delta do Parnaíba*), and searches in virtual herbaria such as JABOT, the *Herbário Virtual da Flora do Brasil*, and the SpeciesLink database. Additional tools included dichotomous keys, species descriptions, and consultation with experts specialized in different botanical families. All collected botanical material was incorporated into the HDELTA herbarium. Taxonomic nomenclature, including author citations and synonyms, was updated using the *Flora e Funga do Brasil* online database (http://floradobrasil.jbrj.gov.br/2021). For species not listed in that database, we consulted The World Flora Online database (http://www.worldfloraonline.org/). Taxonomic organization followed the Angiosperm Phylogeny Group IV (APG IV, 2016), except for the Turneraceae family (Kunth ex DC.), which was maintained as a separate group due to robust evidence supporting its monophyly (Tokuoka, 2012; Thulin et al., 2012).

### Data Analysis

For data analysis, we compiled a master dataset using Microsoft Excel^®^, which included variables such as life phase (pre-reproductive, reproductive, and post-reproductive), age, gender (F for female, M for male), medicinal plant composition and therapeutic target composition for each participant. Vernacular plant names and therapeutic targets were standardized across entries. To avoid redundancy, duplicate mentions of the same plant–therapeutic target pairing—collected via both free listing and photographic checklist—were consolidated into a single citation per participant for analysis.

All analyses involving compositional variation across human life phases or between men and women used a binomial response variable composed of the concatenated plant and therapeutic target names (number of citations, since the same plant can have different therapeutic applications). This approach captured finer variation in the structure and differentiation of medicinal plant knowledge throughout participants’ life histories, thereby providing a more robust foundation for evaluating the role of life history in the construction of LMSs. For hypotheses 2 and 4, which specifically addressed the number of plant species and therapeutic targets, the concatenated variable was not used, and plants and targets were analyzed separately. Distinct statistical models were developed to test each hypothesis.

To test H1—that human life history shapes knowledge of medicinal plants—we applied permutational multivariate analysis of variance (PERMANOVA), with a significance level set at 0.05 and 999 permutations. Models were constructed using the combined plant + therapeutic target composition as the response variable, and life phases as a predictor variable. To select and explore the best models (with the greatest explanatory power), we accessed the interactions between life phases and gender, as well as between age and gender. These analyses allowed us to evaluate whether compositional similarity was higher within than between life phases. This hypothesis is structured to falsify the idea that similarity would occur between groups rather than within them, expecting that individuals at the same life phase would share more items in common.

For this hypothesis, we are considering a well-defined composition of medicinal plants and therapeutic targets as having a high degree of sharing or similarity between individuals at each phase. That is, within the same phase, the similarity is greater, or individuals share more items with each other than with individuals at other life phases.

For testing H2, which examines whether certain medicinal plants and therapeutic targets are strongly associated with specific life phases (especially reproductive and post-reproductive), we used Indicator Species Analysis (ISA), a method originally developed in community ecology to identify species that are characteristic of—i.e., diagnostic indicators of—particular groups of sites (Dufrêne & Legendre, 1997; Cáceres & Legendre, 2009). This analysis was based on presence–absence data for both plant species (Prediction 2), and therapeutic targets (Prediction 3), and aimed to test whether distinct sets are significantly linked to reproductive and post-reproductive phases. This analysis is based on the calculation of the Indicator Value (IV), which assesses the abundance and frequency in the composition of medicinal plants and therapeutic targets. This metric indicates whether a set of the mentioned items is strongly associated with specific groups. For example: a medicinal plant cited by nearly all post-reproductive individuals but rarely by individuals in other life phases would yield a high IV and be identified as an indicator strongly associated with the post-reproductive phase. Although ISA was originally devised to detect indicator species across habitats, the same logic applies here to detect plants and therapeutic targets characteristic of life phases or genders. The models were constructed to test that there is a set of medicinal plants and therapeutic targets strongly associated with the reproductive and post-reproductive phases.

To test H3—whether life history modulates gender roles and leads to differentiated medicinal plant knowledge among men and women in reproductive and post-reproductive periods—we applied additional PERMANOVA models. These analyses tested whether women, relative to men at the same life phases, exhibited broader and more distinct plant and therapeutic target compositions (Predictions 4 and 5). Models were again tested with a significance threshold of 0.05 and 999 permutations and were structured to falsify the notion that plant knowledge is differentiated across genders within the same life phases.

Finally, for testing H4—which posits that specific sets of medicinal plants and therapeutic targets are strongly associated with men and women in reproductive and post-reproductive life phases—we used ISA to measure the abundance and frequency (based on presence/absence, composition) of medicinal plants (P6) and therapeutic targets (P7), allowing the identification of patterns of these items strongly associated with men and women in the aforementioned life phases. The models were designed to falsify the assumption that there is one set of medicinal plants strongly associated with men and another set strongly associated with women in reproductive and post-reproductive phases.

A general overview of the research questions, hypotheses, predictions, variables, and analytical methods is presented in Table 3.

**Table 3:** Summary of questions, hypotheses, predictions, variables and analyses performed

General question	Hypothesis	Prediction(s)	Explanatory variable	Response variable	Analyses executed
What is the role of human life history plays in the construction of LMSs? (Q1)	Human life history shapes knowledge about medicinal plants (H1)	We predict that, at all phases, a set of well-defined knowledge associated with medicinal plants + therapeutic targets is structured (P1)	Human life history (pre-reproductive, reproductive and post-reproductive)	Composition of medicinal plants + therapeutic targets	PERMANOVA
What is the role of life history in structuring a set of knowledge about medicinal plants strongly associated with certain phases of life? (Q2)	Human life history modulates the structuring of the set of medicinal plants strongly associated with certain life phases (H2)	We expect to find that adults in reproductive and post-reproductive phases will exhibit a set of medicinal plants strongly associated with these phases compared to individuals in the pre-reproductive phase, which will not express a set of plants strongly associated with this group (P2).	Human life history (pre-reproductive, reproductive and post-reproductive)	Composition of medicinal plants	Indicator species analysis-ISA
		We also expect that adults in reproductive and post-reproductive phases will express a set of therapeutic targets strongly associated with each of these phases compared to pre-reproductive individuals, which will not express a set of therapeutic targets strongly associated with this group (P3).		Composition of therapeutic targets	
What is the role of gender modulated by human life history in the construction of LMSs? (Q3)	Human life history modulates the role of gender as a predictor for the development and differentiation of knowledge about medicinal plants in LMSs (H3)	We expect to find that individuals in reproductive and post-reproductive phases, women express a broader and well-defined composition of plants + therapeutic targets compared to the knowledge of men who are in the same life phase (P4)	Gender (male or female) modulated by human life history (pre-reproductive, reproductive and post-reproductive)	Composition of medicinal plants + therapeutic targets	PERMANOVA
What is the role of human life history in structuring a set of medicinal plants strongly associated with the genders? (Q4)	There is a set of medicinal plants and therapeutic targets associated with men and women in LMSs (H4)	We expect to find a set of medicinal plants (P5), and therapeutic targets (P6) strongly associated with men and another set strongly associated with women within LMSs	Gender (male or female), and human life history (pre-reproductive, reproductive and post-reproductive)	Composition of medicinal plants (for testing P5) and therapeutic targets (for testing P6)	Indicator species analysis-ISA

All analyses were performed using R Software version 4.4.1 (R Core Team, 2024), with *P* < 0.05, considered statistically significant.

## Results

### LMS in the Franco Rural Community Across the Lifespan

In the Franco rural community, local people know 181 medicinal plant species, distributed across 140 genera and 65 botanical families (see Supplementary Materials). Distinct patterns of knowledge related to medicinal plants and therapeutic targets were observed across different life phases. When analyzing the combination of plant species and therapeutic targets, individuals in the pre-reproductive phase reported a total of 683 unique indications. In comparison, adults in the reproductive and post-reproductive phases reported 686 and 1.007 such combinations, respectively.

In terms of medicinal plant knowledge, individuals in the pre-reproductive phase cited or recognized 139 species. The average number of cited species in this group was 25, with a standard deviation of ± 13.92, ranging from 4 to 91 species. These individuals also mentioned 125 therapeutic targets, with an average of 14 cited targets (± 8.04), ranging from 1 to 46. Participants in the reproductive phase cited 150 medicinal plant species, with an average of 43 species per individual (± 12.53), ranging from 24 to 73. They also cited 149 therapeutic targets, with an average of 31 (± 7.97), ranging from 14 to 47 targets.

In the post-reproductive phase, individuals cited or recognized 169 medicinal plant species. The average number of cited species in this group was 50, with a standard deviation of ± 16.06, ranging from 28 to 87 species. Additionally, they mentioned 179 therapeutic targets, with an average of 32 targets per individual (± 11.43), ranging from 14 to 60. These results reflect a progressive development and diversification of EMPK across the human lifespan. In terms of raw numbers, women cited 1.096 unique plant–therapeutic target combinations, while men cited 1.043.

### Does Human Life History Shape EMPK?

We found no evidence to falsify H1, and therefore accept that human life history plays a significant role in shaping EMPK across the human lifespan (Table 4). The composition of medicinal plants and their associated therapeutic targets was found to be strongly associated with individuals’ life phases. This indicates that different patterns of EMPK are structured throughout the human lifespan and are modulated by life history dynamics, features and flexibility.

**Table 4:** Results of PERMANOVA testing whether the different phases of individuals explain the composition of medicinal plants and therapeutic targets in the Franco rural community, Cocal, Piauí, Northeast Brazil

Variables	Degree Freedom	Sum of Squares	*R*^2^	F	Pr(> F)
Life phase	2	2.530	0.07530	3.5566	0.001***
Gender	1	0.544	0.01619	1.5298	0.0004 ***
Life phase: gender	2	0.647	0.01926	0.9099	0.784
Residuals	84	29.881	0.88925		
Total	89	33.602	1.00000		

Our model incorporating age as an explanatory variable (Table 5) showed that, although age contributes to the structuring of medicinal plant knowledge, it does not explain the observed patterns as effectively as human life history. In other words, human life history provided greater explanatory power than chronological age in predicting variation in the composition of medicinal plant knowledge and therapeutic targets.

**Table 5:** Results of PERMANOVA testing whether the age of individuals explains the composition of medicinal plants and therapeutic targets in the Franco rural community, Cocal, Piauí, Northeast Brazil

Variables	Degree Freedom	Sum of Squares	*R*^2^	F	Pr(> F)
Age	1	1.777	0.05288	4.9652	0.001 ***
Gender	1	0.616	0.01835	1.7226	0.0001 ***
Age: gender	1	0.431	0.01283	1.2043	0.106
Residuals	86	30.778	0.91594		
Total	89	33.602	1.00000		

### Is There a Set of Medicinal Plants and Therapeutic Targets Strongly Associated with Reproductive and Post-reproductive Life Phases?

Our analysis to test H2 revealed the presence of a distinct set of medicinal plants (Table 6) and therapeutic targets (Table 7) strongly associated with the reproductive and post-reproductive phases, but not with the pre-reproductive phase. Specifically, eight plant species were associated with the reproductive phase and 35 with the post-reproductive phase. The 62.5% of the species strongly associated with reproductive phase are cultivated in home gardens, 25% are purchased, and 12.5% are grow in forests. Thus, 87.5% of the species are exotic and 12.5% are native to local vegetation. The 57.14% of the species strongly associated with the post-reproductive phase are found in forests, 37.14% are cultivated in home gardens, and 5.72% are purchased at fairs or markets. Thus, 57.14% are native and 42.86% are exotic.

**Table 6:** Results of ISA analysis testing whether there is a set of plants strongly associated with each life stage in the Franco rural community, Cocal, Piauí, Northeast Brazil

ISA results for plants associated with each life phase				
**Reproductive life phase**				
Vernacular name	**A**	**B**	**stat**	**p-value**
*Coleus barbatus* (Andrews) Benth.	0.4319	0.9524	0.641	0.003 **
*Sesamum indicum* L.	0.4110	1.0000	0.641	0.003 **
*Cymbopogon citratus* (DC.) Stapf	0.4088	1.0000	0.639	0.006 **
*Beta vulgaris* L.	0.4392	0.9048	0.630	0.005 **
*Gossypium herbaceum* L.	0.5945	0.6667	0.630	0.001 ***
*Syzygium aromaticum* (L.) Merr. & L.M.Perry	0.4240	0.8571	0.603	0.014 *
*Jatropha gossypiifolia* L.	0.3905	0.9048	0.594	0.037 *
*Bauhinia* sp.	0.8000	0.1429	0.338	0.038 *
**Post-reproductive life phase**				
Vernacular name	**A**	**B**	**stat**	**p-value**
*Croton blanchetianus* Baill.	0.7118	0.7143	0.713	0.001 ***
*Amburana cearensis* (Allemão) A.C.Sm.	0.4732	0.9286	0.663	0.001 ***
*Hymenaea stigonocarpa* Mart. ex Hayne	0.5173	0.8214	0.652	0.001 ***
*Eleutherine bulbosa* (Mill.) Urb.	0.6982	0.6071	0.651	0.001 ***
*Vachellia farnesiana* (L.) Wight & Arn.	0.5022	0.8214	0.642	0.001 ***
*Eucalyptus globulus* Labill.	0.5281	0.7500	0.629	0.001 ***
*Senna alata* (L.) Roxb.	0.7365	0.5357	0.628	0.001 ***
*Piper tuberculatum* Jacq.	1.0000	0.3929	0.627	0.001 ***
*Coleus amboinicus* Lour.	0.5106	0.7500	0.619	0.003 **
*Xylopia frutescens* Aubl.	0.6145	0.6071	0.611	0.003 **
*Ximenia americana* L.	0.3708	0.9643	0.598	0.048 *
*Curcuma longa* L.	0.3986	0.8929	0.597	0.037 *
*Hymenaea courbaril* L.	0.4904	0.7143	0.592	0.002 **
*Luehea candicans* Mart.	0.6983	0.5000	0.591	0.001 ***
*Anacardium occidentale* L.	0.5387	0.6429	0.588	0.001 ***
*Phyllanthus niruri* L.	0.4263	0.7500	0.565	0.038 *
*Cenostigma nordestinum* Gagnon & G.P.Lewis	0.5412	0.5357	0.538	0.010 **
*Solanum tuberosum* L.	0.6181	0.4643	0.536	0.003 **
*Astronium urundeuva* (M.Allemão) Engl.	0.4980	0.5714	0.533	0.018 *
*Ocimum gratissimum* L.	0.5812	0.4643	0.519	0.008 **
*Persea americana* Mill.	0.4932	0.5357	0.514	0.023 *
*Punica granatum* L.	0.4932	0.5357	0.514	0.014 *
*Operculina hamiltonii* (G.Don) D.F.Austin & Staples	0.8571	0.2857	0.495	0.002 **
*Cecropia glaziovii* Snethl.	0.8542	0.2857	0.494	0.003 **
*Smilax* sp.	0.9111	0.2500	0.477	0.003 **
*Tabernaemontana* catharinensis A.DC.	0.6578	0.3214	0.460	0.014 *
*Handroanthus impetiginosus* (Mart. ex DC.) Mattos	0.6292	0.2857	0.424	0.028 *
*Vicia faba* L.	0.8182	0.2143	0.419	0.011 *
*Senna occidentalis* (L.) Link	0.7485	0.2143	0.400	0.029 *
*Copernicia prunifera* (Mill.) H.E.Morre	0.8798	0.1786	0.396	0.010 **
*Maranta arundinacea* L.	0.6923	0.2143	0.385	0.026 *
*Myroxylon peruiferum* L.f.	0.7895	0.1786	0.375	0.043 *
*Operculina macrocarpa* (L.) Urb.	0.7500	0.1429	0.327	0.049 *
*Ocimum basilicum* L.	1.0000	0.1071	0.327	0.041 *
*Handroanthus serratifolius* (Vahl) S.Grose	1.0000	0.1071	0.327	0.038 *

Signif. codes: 0 ‘***’ 0.001 ‘**’ 0.01 ‘*’ 0.05 ‘.’ 0.1 ‘ ’ 1

**Table 7:** Results of ISA analysis testing whether there is a set of therapeutic targets strongly associated with each life stage in the Franco rural community, Cocal, Piauí, Northeast Brazil

ISA results for therapeutic targets associated with each life phase				
**Reproductive life phase**				
Therapeutic targets	**A**	**B**	**stat**	**p-value**
Calm the nerves	0.53770	0.71429	0.620	0.001 ***
Diarrhea	0.37751	1.00000	0.614	0.035 *
Anemia	0.48235	0.71429	0.587	0.004 **
Severe external inflammation	0.48737	0.57143	0.528	0.021 *
Constipation	0.44717	0.57143	0.505	0.050 *
Impinge	0.51882	0.47619	0.497	0.016 *
Furuncle	0.57921	0.33333	0.439	0.021 *
Heartburn	0.68619	0.23810	0.404	0.033 *
Gas buildup	0.64113	0.23810	0.391	0.040 *
Palmar wart	0.85417	0.14286	0.349	0.021 *
Miudinho de criança	0.85417	0.14286	0.349	0.027 *
Rhinitis	1.00000	0.09524	0.309	0.046 *
**Post-reproductive life phase**				
Therapeutic targets	**A**	**B**	**stat**	**p-value**
Inflammation in the uterus	0.5568	0.7500	0.646	0.001 ***
Inflammation in the ovaries	0.5568	0.7500	0.646	0.001 ***
Cancer	0.5230	0.7857	0.641	0.002 **
Infantile colic	0.5150	0.7857	0.636	0.001 ***
General fever	0.4520	0.8929	0.635	0.002 **
Indigestion	0.3918	0.9286	0.603	0.022 *
Inflammation in general	0.4217	0.8571	0.601	0.019 *
Gastritis	0.6325	0.5357	0.582	0.002 **
Worm	0.6548	0.5000	0.572	0.002 **
Kidney stones	0.4772	0.6786	0.569	0.014 *
General colic	0.5516	0.5000	0.525	0.008 **
Childhood diarrhea	0.8170	0.3214	0.512	0.001 ***
Urinary tract infection	0.4725	0.5357	0.503	0.043 *
Tooth inflammation	0.7693	0.3214	0.497	0.002 **
Fracture	0.7455	0.2857	0.462	0.010 **
Abortion	0.6000	0.2857	0.414	0.047 *
Changes in the menstrual cycle	0.6764	0.2500	0.411	0.017 *
General pain in the belly button	1.0000	0.1429	0.378	0.010 **
Stroke	0.8542	0.1429	0.349	0.035 *
General leg pain	0.8542	0.1429	0.349	0.034 *
Infection in general	0.8542	0.1429	0.349	0.031 *
Bronchitis	1.0000	0.1071	0.327	0.043 *
Stop bleeding in the mouth	1.0000	0.1071	0.327	0.032 *
Clean the uterus (women’s health)	1.0000	0.1071	0.327	0.034 *
Prevent infant colic	1.0000	0.1071	0.327	0.033 *
Rheumatism	1.0000	0.1071	0.327	0.038 *
Thrombosis	1.0000	0.1071	0.327	0.043 *

Signif. codes: 0 ‘***’ 0.001 ‘**’ 0.01 ‘*’ 0.05 ‘.’ 0.1 ‘ ’ 1

Regarding the therapeutic targets, 12 were strongly linked to the reproductive phase and 27 to the post-reproductive phase. The therapeutic targets strongly associated to these phases are associated with the health of children and women, the elderly, and general problems affecting different body systems in women and men of different ages and life phases. These findings support the hypothesis that certain species and therapeutic targets are particularly salient during the reproductive and post-reproductive periods, reinforcing the idea that human life history plays a central role in structuring LMSs across the human lifespan.

### Do Women Have Differentiated EMPK Compared to Men in Reproductive and Post-reproductive Phases?

We found sufficient evidence to reject H3, which proposed that gender roles modulated by life history would result in differentiated knowledge between men and women during the reproductive and post-reproductive phases (Table 8). EMPK differs between men and women only during the reproductive phase. Therefore, although women tended to report broader knowledge of medicinal plants across all life phases, this variation was not sufficient to indicate a significant difference in EMPK between genders when modulated by life history in pre-reproductive and post-reproductive life phases.

**Table 8:** Results of PERMANOVA testing whether differentiated EMPK between women and men in reproductive and post-reproductive phases in the Franco rural community, Cocal, Piauí, Northeast Brazil

	Degree Freedom	Sum of Squares	*R* ^2^	F	Pr(> F)
Pre-reproductive					
Model	1	0.3794	0.02519	1.0077	0.447
Residual	39	14.6844	0.97481		
Total	40	15.039	1.00000		
Reproductive					
Model	1	0.4424	0.06571	1.3363	0.023*
Residual	19	6.2904	0.93429		
Total	20	6.7328	1.00000		
Post-reproductive					
Model	1	0.3627	0.03919	1.0604	0.29
Residual	26	8.8925	0.96081		
Total	27	9.2551	1.00000		

Signif. codes: 0 ‘***’ 0.001 ‘**’ 0.01 ‘*’ 0.05 ‘.’ 0.1 ‘ ’ 1

Although human life history significantly modulates gender roles in relation to medicinal plant knowledge, gender isolated emerged as a key factor explaining broad patterns of medicinal plant knowledge across individuals (Table 4). Gender also explains the differentiation of EMPK when it is not interacting with age (Table 5).

### Is There a Set of Plants and Therapeutic Targets Strongly Associated with EMPK of Men and Women?

Our analysis of H4 revealed that there is a distinct set of medicinal plants and therapeutic targets strongly associated with women, but not with men. Therefore, we find sufficient evidence to reject H4 since there is no set of medicinal plants and therapeutic targets strongly associated with men within the LMS investigated. Specifically, 19 plant species (Table 9) and 18 therapeutic targets (Table 10) were significantly associated with the female gender. Most of these species are cultivated in home gardens (*n* = 15, 78.95%), followed by plants purchased at fairs and/or markets (*n* = 3, 15.79%) and plants that grow spontaneously in anthropized areas (*n* = 1, 5.26%). The therapeutic targets strongly associated with women’s EMPK are not exclusively linked to women’s health, but also to the health of children, the elderly, and people of different ages. These insights suggests that women may be the primary caregivers for people across different genders and age groups.

**Table 9:** Results of ISA analysis testing whether there is a set of plants strongly associated with females and males in the Franco rural community, Cocal, Piauí, Northeast Brazil

ISA results for plants associated with female and males				
Women				
Vernacular name	A	B	stat	*p*-value
*Mentha* ^x^ v*illosa* Huds.	0.5820	0.8462	0.702	0.016 *
*Syzygium aromaticum* (L.) Merr. & L.M.Perry	0.5841	0.7436	0.659	0.048 *
*Ruta graveolens* L.	0.6714	0.6410	0.656	0.003 **
*Carica papaya* L.	0.6579	0.6410	0.649	0.006 **
*Kalanchoe pinnata* (Lam.) Pers.	0.7140	0.5385	0.620	0.003 **
*Punica granatum* L.	0.7440	0.5128	0.618	0.001 ***
*Malpighia emarginata* DC.	0.6931	0.4872	0.581	0.007 **
*Eleutherine bulbosa* (Mill.) Urb.	0.7493	0.4103	0.554	0.005 **
*Allium* sp.	0.6623	0.4615	0.553	0.022 *
*Ocimum gratissimum* L.	0.7658	0.3846	0.543	0.006 **
*Kalanchoe* sp.	0.9290	0.2564	0.488	0.004 **
*Dracaena trifasciata* (Prain) Mabb.	0.7234	0.3077	0.472	0.029 *
*Musa* ^x^ *paradisiaca* L.	0.7658	0.2564	0.443	0.034 *
*Alternanthera brasiliana* (L.) Kuntze	1.0000	0.1795	0.424	0.004 **
*Lavandula angustifolia* Mill.	0.9015	0.1795	0.402	0.010 **
*Senna occidentalis* (L.) Link	0.9015	0.1795	0.402	0.011 *
*Justicia pectoralis* Jacq.	1.0000	0.1282	0.358	0.017 *
*Zamioculcas zamiifolia* (G.Lodd.) Engl.	1.0000	0.1026	0.320	0.041 *
*Jatropha multifida* L.	1.0000	0.1026	0.320	0.028 *

Signif. codes: 0 ‘***’ 0.001 ‘**’ 0.01 ‘*’ 0.05 ‘.’ 0.1 ‘ ’ 1

**Table 10:** Results of ISA analysis testing whether there is a set of therapeutic targets strongly associated with females and males in the Franco rural community, Cocal, Piauí, Northeast Brazil

ISA results for therapeutic targets associated with female and males				
Women				
Therapeutic targets	A	B	stat	*p*-value
Inflammation in the uterus	0.7907	0.6667	0.726	0.001 ***
Inflammation in the ovaries	0.7907	0.6667	0.726	0.001 ***
Anemia	0.7002	0.6410	0.670	0.001 ***
Cancer	0.7002	0.6410	0.670	0.001 ***
Evil eye	0.6915	0.6154	0.652	0.002 **
Infant colic	0.6528	0.5897	0.620	0.012 *
Calm the nerves	0.6727	0.5641	0.616	0.007 **
General colic	0.7969	0.4615	0.606	0.001 ***
Menstrual colic	0.8673	0.3846	0.578	0.001 ***
High blood pressure	0.6992	0.4103	0.536	0.022 *
Earache	0.7824	0.2821	0.470	0.014 *
Abortion	0.7969	0.2308	0.429	0.037 *
General heart problem	0.7969	0.2308	0.429	0.027 *
Tonsillitis	1.0000	0.1795	0.424	0.002 **
Changes in the menstrual cycle	0.8395	0.2051	0.415	0.018 *
Cholesterol	0.8870	0.1538	0.369	0.044 *
Arrhythmia	1.0000	0.1026	0.320	0.037 *
Prevent cancer	1.0000	0.1026	0.320	0.028 *

Signif. codes: 0 ‘***’ 0.001 ‘**’ 0.01 ‘*’ 0.05 ‘.’ 0.1 ‘ ’ 1.

**Fig. 4:**
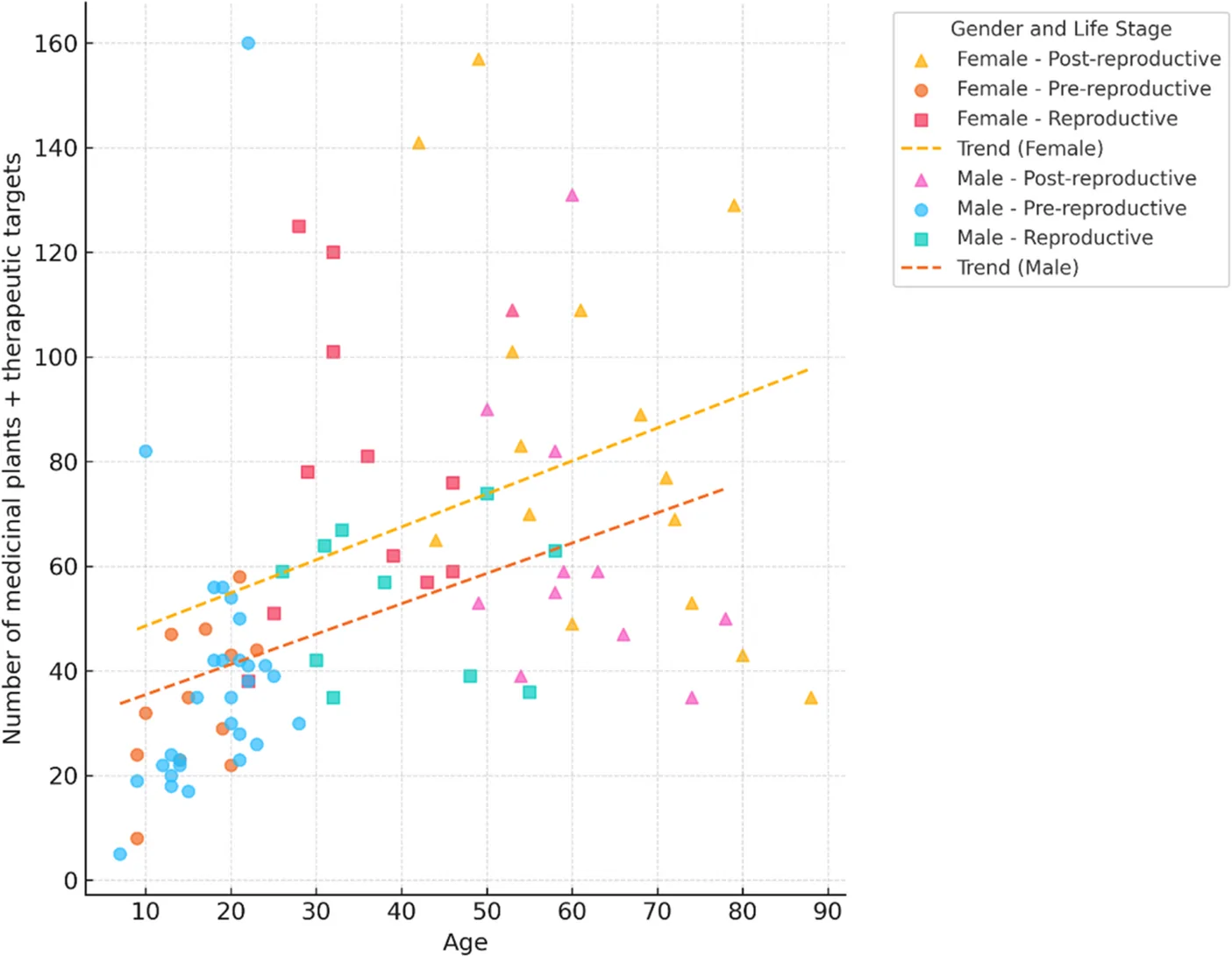
Distribution of the number of citations for the category medicinal plants + therapeutic targets by life phase, gender, and age

The number of the combined category of medicinal plants + therapeutic targets varied between different life phases, genders, and age groups (Fig. 4). In the pre-reproductive phase, girls cited 266 different medicinal plant + therapeutic target combinations. The average number of cited concatenated combination was 33.5, with a standard deviation of ± 13.5, ranging from 8 to 58 citations. The boys at this phase cited 550 different combinations. The average number of cited concatenated combination was 35, with a standard deviation of ± 27.60, ranging from 5 to 160 citations.

Women of reproductive phase cited 484 different medicinal plant + therapeutic target combinations. The average number cited concatenated combination was 76, with a standard deviation of ± 26.80, ranging from 38 to 125 citations. Men at this phase cited 358 different combinations for medicinal plants + therapeutic targets. The average number cited concatenated combinations was 58, with a standard deviation of ± 13.55, ranging from 35 to 74 citations. In the post-reproductive phase, women cited 729 different combinations of medicinal plants and therapeutic targets. The average number cited concatenated combination was 80, with a standard deviation of ± 34.63, ranging 35 to 157 citations. The men at this phase cited 500 combinations of medicinal plants + therapeutic targets. The average was 57 citations, with a standard deviation of ± 28.25, ranging from 39 to 131.

## Discussion

Our findings expand current understanding of the construction of LMSs in several important pathways. Firstly, individuals within the same life phase share a broader set of medicinal plant knowledge than individuals in different life phases, indicating that human life history plays a fundamental and primary role in shaping LMSs. Secondly, there is a distinct set of medicinal plants and therapeutic targets strongly associated with the reproductive and post-reproductive phases, but not with the pre-reproductive phase. This suggests that medicinal plant knowledge becomes more specialized and better structured following the first reproductive event or family formation, a point at which environmental demands and social responsibilities shift markedly from those experienced during the extended human childhood.

Third, we expected to find the structuring or differentiation of EMPK between men and women only in the reproductive and post-reproductive phases, but our findings suggest that EMPK differs between men and women only during the reproductive phase. In a general context, gender isolated explains the composition of medicinal plants and therapeutic targets. This highlights the importance of recognizing that both men and women contribute meaningfully to the construction and maintenance of LMSs across the human lifespan. Finally, our analyses identified a set of medicinal plants and therapeutic targets that are strongly associated with women, but not with men. This pattern suggests a domain of specialized knowledge held by women.

### The Human Life History Shapes EMPK Across the Lifespan

Our findings suggest that human life history provides a robust explanatory framework for understanding how medicinal plant knowledge is structured across the lifespan. At each life phase, individuals share more medicinal plant knowledge with others in the same phase than with those in different or others life phases, indicating that life history plays a central role in the construction of LMSs. The different patterns of knowledge within LMS investigated based on the composition of medicinal plants and therapeutic targets appear to be shaped by life-phase-specific demands, responsibilities, and embodied capital, offering a refined view of how knowledge is acquired and mobilized throughout human development.

These results offer empirical support for theoretical propositions drawn from LHT (Stearns, 1992; Roff, 1993), ECT (Kaplan, 1996), and the GH (Hawkes et al., 1989). Our insights also align with the idea that human beings develop appropriate knowledge to each life phase, with different sets of medicinal plant knowledge being structured in accordance with the ‘functional objectives’ of each life phase (Schniter et al., 2021; Del Giudice et al., 2015). In this way, during the pre-reproductive phase, individuals aim to acquire basic skills and knowledge, including those necessary to support themselves and eventually attract a partner. In the reproductive phase, the focus shifts toward reproduction, child-rearing, and the optimal use of knowledge to ensure the health and development of offspring. Individuals focus on raising children to achieve success and fulfill the different goals of the pre-reproductive and reproductive phases. Individuals in post-reproductive period to contribute to the goals of previous phases within the family context, despite age-related declines in physical capacity (see Schniter et al., 2021).

In the Franco rural community, individuals at different life phases exhibit differences in behavior regarding the use of medicinal plants (Siqueira et al., 2026). Locally, children are exposed to medicinal plants from an early age, often through observation or participation in plant collection and preparation of home remedies alongside adults. This extended phase of life is important for the development and differentiation of knowledge about medicinal plants (Bruschi et al., 2019; Sousa et al., 2022; Gallois et al., 2024). The development of knowledge about medicinal plants begins in childhood and tends to expand, with children and adolescents exhibiting extensive knowledge of this category of use (Gallois et al., 2024). During early childhood and adolescence—phases marked by high dependency and learning—children rarely prepare medicines independently, especially those requiring cooking or specialized skills. However, they do begin to make simple preparations in later childhood and early adulthood, as physical capabilities, cognitive development, and experiential learning converge to allow greater autonomy. Extended human childhood, as noted by scholars (Bogin, 1997; Richerson & Boyd, 2020; Gurven et al., 2020), likely evolved to accommodate the time required to master complex knowledge systems like those involving medicinal plants.

Play is another mechanism through which ecological knowledge is built during childhood. In the Franco rural community, children frequently engage in playful activities in various landscapes—home gardens, different anthropogenic landscape, and forested areas—constructing small shelters, mimicking food preparation, building traps, and pretending to fish. These playful experiences contribute to ecological familiarity, even if direct handling of medicinal preparations (e.g., boiling plants for teas) is limited. Playing with medicinal plants can be a rare phenomenon because it is knowledge that directly deals with health, and is considered a serious issue for the community. On the occasions when we saw children playing with fire and preparing teas, only the plant structures were boiled and they behaved as if they were smelling the tea, but they never drank the preparation. Likewise, when they played at making food with plants, they only prepared the dishes, but never consumed them. In the later years of adolescence, play decreases compared to early childhood (Gallois et al., 2015), and individuals contribute more economically to the family’s subsistence (Kaplan, 1996).

As individuals reach reproductive age, we observe a notable increase in both the breadth and complexity of medicinal plant knowledge. This increase aligns with life history predictions: the first reproductive event marks a shift in priorities, as individuals assume responsibilities for offspring and family health. Adults in the reproductive and post-reproductive phase in the Franco community demonstrate broader and more detailed knowledge than those in earlier phase, and this knowledge is essential for managing a wide range of disease events. During participant observation, adults in these phases were frequently seen cultivating medicinal plants in their home gardens and preparing remedies for family members. Post-reproductive individuals, particularly grandmothers, played a central role in these practices, often acting as reservoirs and transmitters of complex and specialized medicinal knowledge—contributions that likely enhance inclusive fitness (Hawkes et al., 1989; Schniter et al., 2023).

### There is a Set of Medicinal Plants and Therapeutic Targets Strongly Associated with Adults in Reproductive and Post-Reproductive Phases, but not with Pre-Reproductive Individuals

Our findings indicate that there is a distinct set of medicinal plants and therapeutic targets strongly associated with the adults’ EMPK in the reproductive and post-reproductive phases, but not with individuals in the pre-reproductive phase. These results are consistent with predictions from life histories models (Hawkes et al., 1989; Stearns, 1992; Roff, 1993; Kaplan, 1996), which posit that knowledge systems develop in accordance with life-phase-specific challenges and responsibilities (Siqueira et al., 2026).

A key question arises: why do individuals in the pre-reproductive phase not exhibit a corresponding set of medicinal plants and therapeutic targets strongly associated with their life phase? During this period, children are undergoing significant physical, sexual, and cognitive development, and are actively acquiring cultural knowledge that will support them in adulthood (Gallois & Reyes-García, 2018). Human childhood is notably extended, providing time for the acquisition of adaptive knowledge through observation and participation in social practices (Lee, 2013; Barrett et al., 2016). In addition to the intensive learning at this phase, children can also teach others and produce innovations in local knowledge systems (Lew-Levy & Amir, 2024).

Children may develop an extensive knowledge regarding medicinal plants (Gallois et al., 2024), and recognize species not mentioned by adults (Sousa et al., 2022). However, in the Franco rural community, the social role of family healthcare is more associated with adults with children and grandchildren and is not a responsibility of children and adolescents, although they may cooperate in human health-seeking behaviors. As children and adolescents grow and develop, they experience changes in the increase in embodied capital, which may be associated with a broader knowledge about natural resources (Schniter et al., 2021).

However, our results suggest that at this phase individuals are not concerned with structuring specialized knowledge about medicinal plants, and therefore, there is no EMPK strongly associated with this phase. Children at this phase are primarily engaged in learning about therapeutic resources—such as vernacular names, locations of occurrence, plant parts used, preparation methods, administration routes, and contraindications—but do not yet face the same ecological and social pressures as adults in reproductive and post-reproductive phases. According to ECT (Kaplan, 1996), the demands of the pre-reproductive phase are largely self-directed and do not require the same level of embodied knowledge needed for family or community care. Thus, while children and adolescents actively participate in foraging and are exposed to medicinal plant use within the household, they are not responsible for the preparation of remedies—especially those requiring greater physical effort or technical skill. As they grow, however, these individuals progressively assume more responsibility and begin preparing their own remedies, particularly in the later childhood and early adulthood.

In contrast, the reproductive life phase marks a shift toward more complex demands related to self-care and care for dependent offspring. Adults must expand their EMPK to address the health needs of their children and kin (Schniter et al., 2021). This development of knowledge reflects increased embodied capital, structured in response to evolving social and ecological demands. In the post-reproductive phase, while physical and cognitive abilities may decline (Craik & Rose, 2012; Koster et al., 2020), individuals continue to play an important role in maintaining family and community health. According to the GH (Hawkes et al., 1989; Hawkes, 2020), post-reproductive longevity likely evolved to allow older adults, especially grandmothers, to support offspring and grandchildren, facilitating earlier weaning and shorter birth intervals.

This life phase is crucial for the intergenerational transmission of knowledge. Older adults are often the custodians of complex skills and experiential wisdom, especially those that do not require physical strength but are essential for health-related decision-making (Schniter et al., 2023). In the Franco rural community, we observed that older adults often cultivate medicinal plants in their home gardens, and prepare remedies for their children and grandchildren. These individuals are highly engaged in healthcare practices and serve as primary sources of knowledge transmission. Our findings suggest that the knowledge strongly associated with individuals at this phase corresponds to native species that occurring in the local vegetation.

Thus, the presence of a distinct set of medicinal plants and therapeutic targets associated with the reproductive and post-reproductive phases highlights how EMPK is shaped by human life history. At these phases, there is a set of therapeutic targets that encompass different diseases of different body systems, from childhood illnesses to diseases in the elderly, which is important for better responding to the pressures imposed by illness events. These knowledge systems are adapted to the changing demands of each phase and serve to increase therapeutic safety in response to disease events. By contrast, the absence of such structured knowledge in the pre-reproductive phase reflects the developmental role of childhood, wherein individuals are immersed in learning rather than application, phase characterized by intense learning, exploration, and flexibility.

### Differentiated EMPK Between Women and Men

In the Franco rural community, both men and women play crucial roles in the construction, maintenance, and functioning of LMS. Our findings suggest that gender roles, when considered in conjunction with human life history (especially in reproductive and post-reproductive), do not predict significant differences in the composition of EMPK between men and women. EMPK is only differentiated between males and females during the reproductive phase. However, our findings show that the differentiation of EMPK between men and women begins to develop very early in childhood, but only in the reproductive phase is the differentiation well-structured. The pre-reproductive period is an extra phase in human life history, characterized by intense learning and flexibility, and children and adolescents are not subject to the same pressures of family health care as adults (Kaplan, 1996; Richerson & Boyd, 2020). Therefore, it was to be expected that there would be no differences in the composition of the EMPK between boys and girls at this phase.

In the Franco community, from childhood to the post-reproductive phase, there are differences in behavior, and social gender roles change throughout life. Gender-based occupations and the different activities performed by individuals can broaden our understanding of why EMPK differs between men and women only during the reproductive phase (see Schniter et al., 2021). In the local context, from a very young age children play in groups shared by boys and girls. As they get older, they are seen playing less, and when they do, it is usually in groups of boys and groups of girls separately. Play can be important for promoting interactions between children and natural resources, fostering the acquisition of knowledge relevant to survival (Gallois et al., 2015). Play offers new opportunities for learning and exploration (Quinlan et al., 2016). As children grow and mature, there is a distancing from play activities and a transition between play and subsistence activities, with differences between boys and girls, with the latter doing it much earlier (Quinlan et al., 2016).

The differences in occupational niches occupied by men and women, influenced by their occupation, are important for the development and differentiation of knowledge about natural resources across the human lifespan (Schniter et al., 2021). The different occupations based on social roles are important for explaining knowledge about medicinal plants (Alencar et al. 2014a). In the Franco rural community, as children grow and mature, they begin to assume different social gender roles, influenced by various factors such as occupations. Boys tend to take on various occupations associated with the male gender, including hunting, fishing, herding livestock, honey foraging, fence building, and different non-natural activities such as masonry, masonry assistant, carpentry, among others. Girls, on the other hand, tend to take on occupations associated with the female gender, such as cooking food, housekeeping and gardening, caring for domestic animals used for food (pigs, chickens, ducks, etc.), and also perform non-natural activities. Both genders are involved in various nature-related activities, such as agricultural practices; however, within these practices, some activities are more associated with men and others with women. The transition from play to different occupations linked to gender roles can structure different patterns of knowledge about medicinal plants (Quinlan et al., 2016). In the Franco rural community, different gender-based occupations are well-structured or defined in reproductive adulthood, when adults assume different levels of responsibility.

Another important aspect is the practice of cooking, which, although children and adolescents can do, is primarily a task associated with adult life and can broaden knowledge about medicinal plants. Cooking practices among men and women can be important for understanding the role of gender in EMPK. Teixidor-Toneu et al. (2021), in an ethnobotanical study in Marrakech, report that women who cook more frequently have a broader knowledge of medicinal plants, while men who cook more frequently do not have a broader knowledge, especially because they are single, have high levels of schooling, and no children. Married men tend to cook less frequently. In the Franco rural community, cooking is an activity strongly associated with women, but men also do it, albeit less frequently. Future studies may consider it relevant to investigate the role of cooking frequency between genders and its role in the development and differentiation of knowledge between men and women at different life phases.

Another important aspect that can be included within the social gender role is parental investment, which may contribute to differences in the composition of medicinal plants. Women may invest more than men in their children’s knowledge of natural resources. For example, Pretelli et al. (2022) reported that in Tanzania, boys raised by fathers tended to have broader knowledge levels compared to girls, while in children raised by mothers, both boys and girls showed similar levels of knowledge about natural resources, suggesting a parental investment bias. Mothers’ broader knowledge compared to fathers tends to result in higher health rates for their offspring (McDade et al., 2007). In the Franco rural community, the gender-based social role associated with family healthcare begins very early. Girls tend to dedicate time and participate in activities related to the cultivation and care of medicinal plants, as well as helping their mothers care for the health of younger siblings, which can contribute to the expansion and differentiation of the EMPK. In this way, humans are characterized by cooperative breeding systems, which involve extensive allomaternal care—that is, caregiving support provided by individuals other than the biological mother, including other children, extended kin, and non-kin (Sear, 2021). This cooperative model, supported by flexibility in childrearing and caregiving (Page et al., 2021), plays a central role in shaping the development of life history traits and knowledge distribution.

In the Franco rural community, although women at all phases of life tend to possess a broader EMPK compared to men, men also fulfill different roles in the construction of the LMS, mainly during reproductive and post-reproductive life phases. Men were frequently seen collecting plants, especially in forested areas or far from residences, preparing and administering plant-based remedies to individuals of different ages, and even sharing their knowledge with various members of the community, which may have contributed to the structuring of a distinct pattern of knowledge. Although women tend to cite a broader EMPK at all phases of life, men tend to broaden their EMPK at older ages, which may have contributed to the lack of differentiation in knowledge between men and women in the post-reproductive phase.

Understanding the role of gender and the construction of LMSs throughout human life can be a complex task, given the complexity of this variable and its association with other factors. A multifactorial approach is necessary for a better understanding of the role of gender and knowledge regarding medicinal plants. Torres-Avilez and Albuquerque (2017) argue that ethnobotanical approaches to gender roles and local knowledge systems should be multifactorial, addressing different aspects such as gender biology, culture, economy and local ecology, as well as different variables that can modulate different social gender roles, such as age, occupations, income, ethnicity, among others. This approach can reduce gender stereotypes in knowledge about natural resources (Pfeiffer & Butz, 2005).

In our approach, we consider gender to be modulated by life history; we do not directly assess it, but there is a set of characteristics throughout each phase that are important for the social construction of gender and may reflect in the differences in knowledge about medicinal plants between men and women. Pfeiffer and Butz (2005, p. 247) argue that it is important to analyze the role of gender and include variables such as “*gender-specific modes of knowledge transmission; gender-differentiated social networks; gender-based cultural roles and spiritual taboos that influence beliefs and social norms regarding each sex’s involvement with different components of managed and unmanaged ecosystems; patterns of marital [re]location; gender differences in access to natural resources; and sex-based differences in access to formal and external knowledge*.”

In addition to these factors, it is important to investigate the time allocated to gender-related activities, the transition between play and the development of different livelihood activities between boys and girls, allomaternal care, parental investment, extended family, and cooperation (who collects the plants, who prepares them, who manages them, etc.) within human health seeking-behavior. These elements may hold the key to a deeper understanding of how gender roles are enacted and how knowledge is maintained or transformed within small-scale societies. Another important aspect is to investigate the characteristics of the knowledge associated with men and women, which includes the origin of the species (native or exotic), the location of access (e.g., home gardens, forests, anthropized areas, etc.), the growth pattern of the species, the therapeutic targets strongly associated with and exclusive to the knowledge of men and women, as well as the plants that are strongly associated with and exclusive to each gender.

These different, meticulous approaches can broaden our understanding of the social role of gender and the construction of LMSs, particularly due to contrasting insights in different parts of the world. In different contexts, measured by the number of species, women have a broader knowledge of medicinal plants (Tng et al., 2021; Tinitana et al., 2025), while in many others men possess a broader knowledge (Albuquerque et al., 2011; Tadesse et al., 2025), although there may also be no variation in the knowledge of men and women (Silva et al., 2011; Weckmüller et al., 2019; Souza et al., 2021). In a systematic review on the role of gender and the construction of LMSs, Torres-Avilez et al. (2016) suggest that there are knowledge patterns that differ between men and women on a global scale, and in continental analysis there are continents where men have a broader knowledge (Africa, for example), while in others women have a broader knowledge (such as in America). These differences can also vary between countries; on smaller scales, the differences in knowledge between men and women are much more evident. In general, studies that focus on understanding the role of gender and the construction of LMSs have used the number of plants as a proxy, and often richness may not provide details about the differences in knowledge between men and women. Gomes et al. (2024) in Northeast Brazil recorded that when considering the richness of species cited between men and women, there are no significant differences in knowledge, but when the analysis is based on plant composition, different patterns of knowledge are structured between genders.

### There is a Set of Medicinal Plants and Therapeutic Targets Strongly Associated with Women, but not with Men

Our results indicate that human life history modulates gender roles in a way that produces differentiated EMPK across all life phases. Although both men and women contribute to the construction of LMSs, we found a set of medicinal plants and therapeutic targets that is strongly associated with women, but no equivalent pattern for men at any life phase. Therefore, H4 was falsified, as it predicted such associations for both genders. This outcome reinforces that women can play different active roles in the LMS, especially by having specialized knowledge.

This pattern of behavior can begin very early in childhood, with girls expanding their knowledge of plants relevant to healthcare compared to boys, generally demonstrating a broader EMPK (Quinlan et al., 2016). In the Franco rural community, when a child becomes ill, the primary responsibility for care typically falls to the parents, but grandparents—especially grandmothers—along with siblings and other adults, frequently contribute to managing health-related needs. Girls and women, in particular, are often the first to initiate health-seeking behaviors during disease episodes. These observed patterns may contribute to the structuring of a set of therapeutic targets treated with medicinal plants strongly associated with women’s knowledge. Mothers appear to invest more than fathers in their local botanical knowledge, which may enhance adaptive advantages and improve children’s health outcomes (McDade et al., 2007). These differences in parental investment may be important for structuring a set of plants and therapeutic targets strongly associated with women.

Women can share more of their knowledge about plants than men, also structuring a more cohesive network with more central individuals, making the network more cohesive (Costa et al., 2021). In the Franco rural community, women sharing more items themselves may contribute to structuring the pattern of EMPK strongly associated with them.

Although women may possess extensive knowledge for dealing with diseases related to women’s health (see Towns & van Andel, 2014; Khadin et al., 2023; Ramos et al., 2025), they may also possess knowledge strongly associated with and linked to individuals’ health of different phases, genders, and ages. Our results show that there are diseases linked to children’s health that are strongly associated with women, which is important for maintaining health during the long human childhood. Knowledge about plants for children’s health is an important component of LMSs (Petran et al., 2024; Ullah et al., 2025). These dynamics position women as key actors in socio-ecological systems, with clear implications for child health.

In this way, mothers’ plant knowledge has been positively correlated with improved child health outcomes, including lower infection rates and better general well-being (McDade et al., 2007; Miller, 2011; Tanner et al., 2011). Moreover, mothers often possess a specialized body of knowledge focused on children’s healthcare (Towns et al., 2014), which is vital not only for offspring survival but also for their developmental success, reproductive potential, and long-term fitness (Schniter et al., 2021). Women’s medicinal plant knowledge also plays a critical role in confronting broader environmental and health challenges, including malnutrition and its associated diseases (Vissoh et al., 2024). In this way, diseases associated with childhood health can establish patterns of knowledge strongly associated with women rather than men (Souza et al., 2021).

In addition to therapeutic targets linked to women’s and children’s health, many of the therapeutic targets strongly associated with EMPK in women are related to diseases affecting post-reproductive adults and people of different ages, including men. Numerous studies have highlighted the vital role that women play in the construction of LMSs throughout the lifespan, attributing this to their central involvement in household and family healthcare (Teklehaymanot, 2009; Almeida et al., 2012). In the Franco rural community, when a family member becomes ill, it is usually the women—mothers or grandmothers—who are the first to act, initiating the collection and preparation of medicinal plants. This proactive role reinforces the structuring of a set medicinal plants + therapeutic targets strongly associated and specialized with women in the reality investigated.

These gendered patterns of knowledge likely reflect broader social and occupational roles. Women’s engagement in family healthcare situates them within a specific socioecological niche, which fosters the development of differentiated knowledge systems (Voeks, 2007; Camou-Gerrero et al., 2008). Men and women occupy distinct roles and responsibilities within the community, and these occupational niches shape how each group interacts with natural resources. In this way, gendered roles contribute to the structure and intravariation of local ecological knowledge (Schniter et al., 2021), and are fundamental to the collective construction of LMSs (Torres-Avilez et al., 2024).

Another important aspect is that most of the species strongly associated with women are cultivated in home gardens. These spaces are relevant for the people’s therapeutic safety, and also for resilience, sustainability, adaptation, and evolution of LMSs (Pala et al., 2019; Roy et al., 2022; Silva et al., 2023; Hou et al., 2024; Konthoujam & Meitei, 2025). Different lines of evidence have suggested that women are more familiar with plant species associated with home gardens than men, while men are more familiar with plants associated with environments far from homes (Kutal et al., 2021; Tng et al., 2021; Santana et al., 2022; Gomes et al., 2024). In the Franco rural community, the primary role of maintaining the home gardens belongs to women; they spend more time than men caring for the plants and cultivate a wide variety of medicinal plants in these spaces (Siqueira et al. 2017, 2020a, b), and this seems to be a pattern in different areas around the world (see Díaz-Reviriego et al., 2015; González-Ball et al., 2022).

Fifteen species strongly associated with women’s knowledge are cultivated in home gardens and three are purchased at markets; since they do not grow spontaneously or are part of the local vegetation, we consider them exotic. Only one species grows spontaneously in anthropized areas. Exotic species play important roles in structuring LMSs, being important for increasing redundancy and the capacity to absorb disturbances in these systems, also filling therapeutic gaps where there are no native species (Alencar et al. 2014b), and they reduce the use pressure on native species that share the same applications within the LMSs (Silva et al., 2014). Cultivating exotic medicinal plants can have different adaptive advantages and influence human behavior regarding the selection and inclusion of this resources into LMSs (Gama et al., 2018).

### Limitations

Our study employed theoretical frameworks from LHT, ECT, and the GH to define a set of characteristics and behaviors associated with pre-reproductive, reproductive, and post-reproductive phases. These life phases served as the basis for constructing our explanatory variable, enabling us to investigate how EMPK is structured across the human lifespan. However, several limitations should be noted below.

Firstly, our hypotheses were not explicitly designed to investigate the relationship between medicinal plant knowledge and the trade-offs inherent in human life history—a key focus within LHTs (Stearns, 1992; Roff, 1993). While we drew from concepts within these theories, we did not directly assess how knowledge acquisition might interact with energetic trade-offs or resource allocation, which remains a gap in advancing life history models (see Sear, 2020) within evolutionary ethnobiology.

Secondly, our classification of individuals into three discrete life phases may have obscured intra-phase heterogeneity. Each phase encompasses a range of ages, and individuals within the same phase can possess varying levels of knowledge. For example, the pre-reproductive phase includes first and second childhood, the juvenile phase, and adolescence (Bogin, 1997), each marked by distinct developmental milestones. Similarly, in the reproductive phase, the demands of offspring may diminish as children become more self-sufficient, potentially altering parental investment. Other relevant aspect is that post-reproductive individuals, though no longer reproductively active, may still be heavily involved in caregiving and resource provision for children and grandchildren.

Our emphasis on composition—specifically the diversity of medicinal plants + therapeutic targets combinations—limits our ability to capture developmental trajectories or rates of knowledge acquisition. Future research should complement our approach with analyses of knowledge development over time (e.g., Pretelli et al., 2022), which would allow for more nuanced interpretations of how EMPK evolves within human lifespan. Furthermore, longitudinal designs or studies with spatial replications can provide new insights into the role of life history and construction of LMSs by human species.

Additionally, although we applied accuracy-enhancing procedures and combined data from free listing with photographic checklist, all ethnobiological techniques are subject to potential biases. Memory-based techniques, such as free listing, may underrepresent knowledge in certain age groups. However, the combination of recall and recognition-based methods, alongside careful data validation, helped mitigate these risks.

## Conclusions

Our findings demonstrate that life history events offer greater explanatory power than age in predicting EMPK across the human lifespan. In the Franco rural community, medicinal plants play a vital role in primary healthcare (Siqueira et al. 2017, 2020a, b), and investing time and energy in acquiring EMPK appears to be an adaptive strategy for promoting health, survival and reproductive success.

Our results suggest that knowledge about medicinal plants is not static but develops and differentiates in alignment with life-phase-specific responsibilities and environmental pressures, such as disease events. While other primates are known to use medicinal plants (De la Fuente et al., 2022), only humans have constructed complex, culturally transmitted LMSs. The unique features of human life history—including extended childhood, cooperative breeding, and prolonged post-reproductive longevity—appear to be key drivers in the emergence and refinement of these systems.

We have shown that there is a set of medicinal plants and therapeutic targets strongly associated with the reproductive and post-reproductive phases. By focusing on reproductive transitions and caregiving roles—as proposed by Stearns (1992), Kaplan (1996), and Hawkes et al. (1989)—we have expanded the conceptual framework used to study LMSs. This approach offers a powerful lens for understanding the social, ecological, and evolutionary dynamics that shape medicinal plant knowledge within population level.

These insights also carry important implications for conservation. Because each life phase is associated with a distinct set of medicinal plants and therapeutic targets, conservation strategies must incorporate knowledge held by individuals across the lifespan—from children to elders. Neglecting the contribution of any age group risks undermining the effectiveness and sustainability of conservation efforts.

Finally, our results show that there is a differentiation in EMPK between men and women only in the reproductive phases, when adults assume different roles based on gender. In addition, we did find a set of plants and therapeutic targets strongly associated with women, reinforcing their central role in health-related decision-making and family care within LMSs.

## Supplementary Information

Below is the link to the electronic supplementary material.

Supplementary Material 1 (DOCX 86.8 KB)

## Data Availability

The data used to support the findings of this study are available from the corresponding author upon reasonable request.

## Footnotes

1 Knowledge, skills, and abilities in the use of medicinal plants can be considered a form of embodied capital (Kaplan et al., 2003). Quinlan et al. (2016) used the term “*embodied local ethnobotanical knowledge*” to refer to individuals’ knowledge about plants from an ethnobotanical and embodied capital perspectives. In the present investigation, we applied the term “*embodied medicinal plant knowledge*” to refer to the knowledge, skills, competencies, abilities, and practices on the use of medicinal plants from an embodied capital perspective within LMSs.

2 Exclusive and strongly associated are two different pathways. We are considering the set of medicinal plants and therapeutic targets strongly associated with the phases as the knowledge about medicinal plants that is strongly recognized with each group, that is, that was frequently cited and shared among individuals at each phase. Species and therapeutic targets strongly associated with a given phase may not be exclusive to that same phase, since they may also be known by people at other phases.

3 We do not expect to find this same pattern for girls in the pre-reproductive phase because knowledge about medicinal plants is intensively developing, although differentiation begins early (see Pretelli et al., 2022). Also, in adulthood this knowledge is already well structured and differentiated (see Schniter et al., 2021). Furthermore, in the phases after the first reproduction, individuals are subjected to new pressures and demands arising from the demands of their offspring (Hawkes et al., 1989; Hawkes, 2003, 2020; Stearns, 1992; Roff, 1993; Kaplan, 1996). The ecological and evolutionary goals of life in the reproductive and post-reproductive phases are also much broader than those of the pre-reproductive phase (see Schniter et al., 2015, 2021).
