# Supplementary material for: Human Life History is Shaping Embodied Medicinal Plant Knowledge Across the Lifespan
Source: Hum Nat. 2026 Jul 8;37(3):305–52. doi: 10.1007/s12110-026-09527-y (PMC13633173; doi:10.1007/s12110-026-09527-y)
Supplement: Supplementary file 1 [file 12110_2026_9527_MOESM1_ESM.docx]

**SUPPLEMENTARY MATERIALS**

**Table 1** Medicinal plants throughout the life stages in the Franco rural community, Cocal, Piauí, NE Brazil. Conventions: NC: number of citations; NP: number of people who cited the species; SP: gender of the participant, H-man, M-woman

|  | **Botanical family/Scientific name** | **Vernacular name** | **Therapeutic targets** | **Life phases** | **NC** | **NP** | **SP** |
| --- | --- | --- | --- | --- | --- | --- | --- |
| 1. | **ACANTHACEAE** |  |  |  |  |  |  |
| 1. | *Justicia pectoralis* Jacq. | Anador | General body aches, general fever, general headache, general malaise | Reproductive, post-reproductive | 05 | 06 | M |
| 2. | **AMARANTHACEAE** |  |  |  |  |  |  |
| 2. | *Alternanthera brasiliana* (L.) Kuntze | Tretleca | Healing wounds, general headache, general body pain | Reproductive, post-reproductive | 03 | 03 | M |
| 3. | *Beta vulgaris* L. | Beterraba | Flu | Reproductive | 02 | 02 | H, M |
| 4. | *Dysphania ambrosioides*  (L.) Mosyakin & Clemants | Mastruz, mentruz | Calm the nerves, general allergy in the body, anemia, muscle and body pain due to intense work, gallstones, cancer, indigestion, general pain in the body, general pain in the kidneys, fracture, gastritis, flu, inflammation of the tonsils, inflammation in general, blow, pneumonia, healing wounds, worms | Pre-reproductive, reproductive, post-reproductive | 88 | 51 | H, M |
| 5. | *Gomphrena* *globosa* L. | Perpétua | She only knows that the resource is medicine | Post-reproductive | 01 | 01 | M |
| 3. | **AMARYLLIDACEAE** |  |  |  |  |  |  |
| 6. | *Allium cepa* L. | Cebola | Flu | Pre-reproductive, reproductive, post-reproductive | 04 | 04 | H, M |
| 7. | *Allium sativum* L. | Alho | Calm the nerves, general colic, infant colic, coronavirus, infant diarrhea, general body pain, migraine, gas, flu, evil eye, prevent flu, sinusitis | Pre-reproductive, reproductive, post-reproductive | 27 | 21 | H, M |
| 8. | *Allium* sp. | Cebolinha | Bronchitis, flu, cold, sinusitis | Pre-reproductive, reproductive, post-reproductive | 13 | 09 | H, M |
| 9. | *Griffinia gardneriana* (Herb.) Ravenna | Flor-de-trovão | Acne | Pre-reproductive | 01 | 01 | H |
| 10. | *Zephyranthes sylvatica*    (Mart. ex Schult. & Schult.f.) Baker | Flor-de-trovão | Acne | Pre-reproductive | 01 | 01 | H |
| 4. | **ANACARDIACEAE** |  |  |  |  |  |  |
| 11. | *Anacardium occidentale* L. | Cajueiro | Vaginal discharge, stopping bleeding in the mouth, chilblains, mouth infection, throat infection, general inflammation, tooth inflammation, uterus inflammation, ovarian inflammation, wound healing | Pre-reproductive, reproductive, post-reproductive | 20 | 14 | H, M |
| 12. | *Astronium* sp. | Aroeira, gonçalave | Uterus inflammation, ovarian inflammation | Post-reproductive | 02 | 01 | M |
| 13. | *Astronium urundeuva*  (M.Allemão) Engl. | Aroeira | Toothache, general body pain, general fever, flu, general infection, urinary infection, general inflammation, tooth inflammation, uterus inflammation, ovarian inflammation, cleaning the uterus, woman who wants to lose (beginning of miscarriage), measles, healing wounds, | Pre-reproductive, reproductive, post-reproductive | 52 | 36 | H, M |
| 14. | *Mangifera indica* L. | Manga | Diabetes, flu | Pre-reproductive, post-reproductive | 02 | 02 | H, M |
| 15. | *Spondias purpurea* L. | Siriguela | Severe external inflammation, gastritis, evil eye, quebrante | Pre-reproductive, reproductive, post-reproductive | 05 | 03 | H, M |
| 5. | **ANNONACEAE** |  |  |  |  |  |  |
| 16. | *Annona muricata* L. | Graviola | Cancer, cholesterol, diabetes, belly button pain in children, flu, obesity, prevent cancer | Pre-reproductive, reproductive, post-reproductive | 18 | 15 | H, M |
| 17. | *Annona squamosa* L. | Ata | Indigestion, diarrhea | Reproductive, post-reproductive | 04 | 04 | H, M |
| 18 | *Xylopia frutescens* Aubl. | Imbiriba | General colic, menstrual colics, general body pain, general fever, gas buildup | Reprodutctive, post-reproductive | 06 | 05 | M |
| 6. | **APIACEAE** |  |  |  |  |  |  |
| 19. | *Cuminum cyminum* L. | Cumim | General colic, evil eye | Post-reproductive | 02 | 01 | M |
| 20. | *Daucus carota* L. | Cenoura | Obesity | Reproductive | 01 | 01 | M |
| 21. | *Foeniculum vulgare* Mill. | Erva-doce | Calm the nerves, arrhythmia, general colic, infant colic, indigestion, flu, stomach inflammation, general malaise, prevent infant colic | Pre-reproductive, reproductive, post-reproductive | 14 | 14 | H, M |
| 22. | *Petroselinum crispum*  (Mill.) Fuss | Salsa | Inflammation in general, obesity | Pre-reproductive, post-reproductive | 02 | 02 | H, M |
| 7. | **APOCYNACEAE** |  |  |  |  |  |  |
| 23. | *Calotropis procera*  (Aiton) W.T.Aiton | Ciúme | Impinge | Reproductive | 01 | 01 | M |
| 24. | *Catharanthus roseus*  (L.) G.Don | Boa-noite | General allergy in the body | Pre-reproductive | 01 | 01 | H |
| 25. | *Himatanthus drasticus*  (Mart.) Plumel | Janaguva | General body pain, gastritis, dislocation, ulcer | Pre-reproductive, reproductive, post-reproductive | 05 | 05 | H, M |
| 26. | *Tabernaemontana* catharinensis A.DC. | Grão-de-bode | Impinge | Post-reproductive | 02 | 02 | H, M |
| 8. | **ARACEAE** |  |  |  |  |  |  |
| 27. | *Zamioculcas zamiifolia*  (G.Lodd.) Engl. | Miocuca | Evil eye | Post-reproductive | 01 | 01 | M |
| 9. | **ARECACEAE** |  |  |  |  |  |  |
| 28. | *Astrocaryum vulgare* Mart. | Tucum | Flu | Pre-reproductive | 01 | 01 | H |
| 29. | *Bactris gasipaes* Kunth | Pupunha | Healing wounds, removing foreign bodies, general swelling of the body | Reproductive | 02 | 02 | H, M |
| 30. | *Cocos nucifera* L. | Coco, coco-vermelho, coco-amarelo | General allergy in the body, cancer, diarrhea, childhood diarrhea, healing wounds | Pre-reproductive, reproductive, post-reproductive | 06 | 06 | H, M |
| 31. | *Copernicia prunifera*  (Mill.) H.E.Morre | Carnaúba | Asthma, flu, inflammation of the uterus, inflammation of the ovaries, snake bite | Pre-reproductive, post-reproductive | 05 | 04 | H, M |
| 32. | *Euterpe precatoria* Mart. | Açaí | Anemia | Reproductive | 01 | 01 | H |
| 10. | **ARISTOLOCHIACEAE** |  |  |  |  |  |  |
| 33. | *Aristolochia birostris* Duch. | Jericó | Severe external inflammation | Reproductive, post-reproductive | 03 | 03 | H, M |
| 11. | **ASPARAGACEAE** |  |  |  |  |  |  |
| 34. | *Aloe vera*  (L.) Burm.f. | Babosa | Acne, general skin allergy, alleviate scars, cancer, dandruff, Severe external inflammation, chilblains, general weakness, gastritis, flu, hemorrhoids, general inflammation, inflammation in the uterus, inflammation in the ovaries, skin blemishes, melasma, hair growth in patches, hair loss, burns, scratches in the uterus, healing wounds, scabies, worms | Pre-reproductive, reproductive, post-reproductive | 40 | 20 | H, M |
| 35. | *Dracaena trifasciata* (Prain) Mabb. | Espada-de-São-Jorge | Evil eye | Post-reproductive | 01 | 01 | M |
| 12. | **ASTERACEAE** |  |  |  |  |  |  |
| 36. | *Acanthospermum hispidum* DC. | Cabeça-chata | Flu | Reproductive, post-reproductive | 03 | 03 | H, M |
| 37. | *Acmella* sp. | Agrião | Flu, general malaise | Post-reproductive | 02 | 02 | H, M |
| 38. | *Gymnanthemum amygdalinum* (Delile) Sch.Bip. ex Walp. | Alumão, alumã | General colic, indigestion, diarrhea, general body aches | Reproductive, post-reproductive | 07 | 05 | M |
| 39. | *Helianthus annuus* L. | Girassol | Stroke | Pre-reproductive, post-reproductive | 02 | 02 | H, M |
| 40. | *Matricaria chamomilla* L. | Camomila | Calm nerves, anxiety, flu, general sore throat | Pre-reproductive, reproductive, post-reproductive | 08 | 07 | H, M |
| 13. | **BIGNONIACEAE** |  |  |  |  |  |  |
| 41. | *Fridericia platyphylla*  (Cham.) L.G.Lohmann | Mofumbo, mofumbo-branco, mofumbo-amarelo, mofumbo-de-São-José | Indigestion, diarrhea, stopping bleeding, gastritis, flu, general malaise | Pre-reproductive, reproductive, post-reproductive | 08 | 07 | H, M |
| 42. | *Handroanthus impetiginosus*  (Mart. ex DC.) Mattos | Pau-d’arco-roxo | General allergy in the body, flu, general inflammation, inflammation in the uterus, inflammation in the ovaries, healing wounds | Reproductive, post-reproductive | 08 | 05 | H, M |
| 43. | *Handroanthus serratifolius*  (Vahl) S.Grose | Pau-d’arco-amarelo | Flu, cancer | Post-reproductive | 02 | 02 | H, M |
| 14. | **BIXACEAE** |  |  |  |  |  |  |
| 44. | *Bixa orellana* L. | Corante | Asthma | Reproductive | 01 | 01 | M |
| 15. | **BRASSICACEAE** |  |  |  |  |  |  |
| 45. | *Brassica oleracea* L. | Couve-folha, couve-flor, couve | Inflammation in general, obesity | Pre-reproductive, reproductive, post-reproductive | 05 | 05 | H, M |
| 16. | **BROMELIACEAE** |  |  |  |  |  |  |
| 46. | *Ananas comosus*  (L.) Merril | Abacaxi | Kidney stones | Post-reproductive | 02 | 02 | H, M |
| 17. | **BURSERACEAE** |  |  |  |  |  |  |
| 47. | *Commiphora leptophloeos*  (Mart.) J.B.Gillett | Imburana-de-espinho | Child allergy due to heat, menstrual colics, flu, urinary tract infection, inflammation in the mouth, inflammation in the tongue, inflammation in the uterus, inflammation in the ovaries, cleaning the uterus, healing wounds | Pre-reproductive, reproductive, post-reproductive | 34 | 17 | H, M |
| 18. | **CACTACEAE** |  |  |  |  |  |  |
| 48. | *Cereus jamacaru* DC. | Mandacaru | Inflammation in the uterus, inflammation in the ovaries | Post-reproductive | 01 | 01 | M |
| 49. | *Melocactus* *zehntneri* (Britton & Rose) Luetzelb. | Cabeça-de-frade | Evil eye | Pre-reproductive | 01 | 01 | H |
| 19. | **CANNABACEAE** |  |  |  |  |  |  |
| 50. | *Cannabis sativa* L. | Maconha | He only knows that the resource is medicine | Pre-reproductive | 02 | 02 | H |
| 20 | **CARICACEAE** |  |  |  |  |  |  |
| 51. | *Carica papaya* L. | Mamão | Alleviate scarring, indigestion, diarrhea, general hoarseness | Pre-reproductive, reproductive, post-reproductive | 05 | 05 | H, M |
| 21. | **CARYOCARACEAE** |  |  |  |  |  |  |
| 52. | *Caryocar coriaceum* Wittm. | Pequi | Flu | Post-reproductive | 01 | 01 | M |
| 22. | **CLEOMACEAE** |  |  |  |  |  |  |
| 53. | *Tarenaya longicarpa*  Soares Neto & Roalson | Mussambê | Flu, urinary tract infection, prostate inflammation, uterus inflammation, ovarian inflammation | Pre-reproductive, reproductive, post-reproductive | 19 | 18 | H, M |
| 23. | **CLUSIACEAE** |  |  |  |  |  |  |
| 54. | *Platonia insignis* Mart. | Bacuri | General colic | Post-reproductive | 01 | 01 | M |
| 24. | **COMBRETACEAE** |  |  |  |  |  |  |
| 55. | *Combretum leprosum* Mart. | Mofumbo, mofumbo-branco, mofumbo-de-Jesus, mofumbo-de-São-José | Diarrhea, general body pain, stopping bleeding, flu, urinary tract infection, general inflammation, healing wounds | Pre-reproductive, reproductive, post-reproductive | 13 | 11 | H, M |
| 56. | *Combretum mellifluum*  Eichler | Mofumbo-amarelo | Urinary tract infection, flu | Pre-reproductive | 02 | 01 | H |
| 57. | *Combretum* sp. | Mofumbo-vermelho, mofumbo-de-São-José | Indigestion, gastritis, flu, urinary tract infection, inflammation in general | Pre-reproductive, reproductive, post-reproductive | 08 | 05 | H, M |
| 58. | *Terminalia catappa* L. | Castanhola | Diarrhea, general headache, cancer | Pre-reproductive, post-reproductive | 04 | 04 | H |
| 25. | **CONVOLVULACEAE** |  |  |  |  |  |  |
| 59. | *Ipomoea asarifolia*  (Desr.) Roem. & Schult. | Salsa | General inflammation, inflammation of the uterus, inflammation of the ovaries, healing wounds, general dizziness | Pre-reproductive, reproductive | 06 | 05 | H, M |
| 60. | *Ipomoea batatas*  (L.) Lam. | Batata-doce | Anemia | Post-reproductive | 01 | 01 | H |
| 61. | *Operculina hamiltonii*  (G.Don) D.F.Austin & Staples | Batata-de-purga, jalapa | Signs of stroke, general headache, general fever, flu, hemorrhoids, general inflammation, prostate inflammation, uterus inflammation, ovarian inflammation, worms | Reproductive, post-reproductive | 13 | 07 | H, M |
| 62. | *Operculina macrocarpa*  (L.) Urb. | Batata-de-purga, jalapa | Signs of stroke, general headache, general fever, flu, hemorrhoids, general inflammation, prostate inflammation, uterus inflammation, ovarian inflammation, worms | Reproductive, post-reproductive | 13 | 07 | H, M |
| 26. | **CRASSULACEAE** |  |  |  |  |  |  |
| 63. | *Kalanchoe pinnata*  (Lam.) Pers. | Malva-coronha, coroima, | Cancer, diarrhea, flu, childhood flu, inflammation in general, inflammation in the uterus, inflammation in the ovaries | Pre-reproductive, reproductive, post-reproductive | 21 | 15 | H, M |
| 64. | *Kalanchoe* sp. | Arantu | Cancer | Post-reproductive | 01 | 01 | M |
| 27. | **CUCURBITACEAE** |  |  |  |  |  |  |
| 65. | *Citrullus lanatus*  (Thunb.) Matsum. & Nakai | Melancia | Urinary tract infection | Pre-reproductive | 01 | 01 | H |
| 66. | *Cucumis anguria* L. | Maxixe | Clean skin blemishes | Reproductive | 01 | 01 | M |
| 67. | *Cucumis sativus* L. | Pepino | Flu, pneumonia | Reproductive | 02 | 01 | M |
| 68. | *Doyerea* *emetocathartica*  Grosourdy | Batata-de-tiú, tiú, cabeça-de-tiú | Calm nerves, general body pain, general bone pain, snake bite | Pre-reproductive, reproductive, post-reproductive | 07 | 07 | H, M |
| 69. | *Lagenaria siceraria*  (Molina) Standl. | Cabaça | Chilblains | Reproductive, post-reproductive | 02 | 02 | M |
| 70. | *Luffa operculata*  (L.) Cogn. | Bucha-paulista | General body aches, flu | Post-reproductive | 02 | 01 | H |
| 71. | *Momordica charantia* L. | Melão-de-São-Caetano, melão-do-mato | General allergy in the body, diabetes, malaria | Pre-reproductive, reproductive, post-reproductive | 04 | 04 | H, M |
| 28. | **DILLENIACEAE** |  |  |  |  |  |  |
| 72. | *Curatella americana* L. | Janaguba, sambaíba, | Changes in the menstrual cycle, carne rasgada, indigestion, inflammation in the uterus, inflammation in the ovaries | Reproductive, post-reproductive | 08 | 06 | H, M |
| 29. | **EUPHORBIACEAE** |  |  |  |  |  |  |
| 73. | *Cnidoscolus urens*  (L.) Arthur | Cansanção, cansanção-branco | Cancer, toothache | Pre-reproductive, post-reproductive | 03 | 03 | H, M |
| 74. | *Cnidoscolus vitifolius*  (Mill.) Pohl | Cansanção, cansanção-branco | Cancer, toothache, prevent cancer | Pre-reproductive, reproductive, post-reproductive | 08 | 08 | H, M |
| 75. | *Croton blanchetianus* Baill. | Marmeleiro-preto, marmeleiro-roxo | Indigestion, diarrhea, flu | Pre-reproductive, reproductive, post-reproductive | 18 | 16 | H, M |
| 76. | *Croton grewioides* Baill. | Canela-de-cunhã | Calm the nerves, chicken pox, earache, general body pain, migraine, general fever, flu, general inflammation | Pre-reproductive, reproductive, post-reproductive | 17 | 14 | H, M |
| 77. | *Croton heliotropiifolius* Kunth | Velame | Wart, impinge | Reproductive | 02 | 02 | H, M |
| 78. | *Euphorbia tirucalli* L. | Cachorro-pelado | Cancer, gastritis | Post-reproductive | 03 | 03 | H, M |
| 79. | *Jatropha curcas* L. | Pinhão-manso, pinhão-branco | Vaginal bacteria, cancer, stroke, stop bleeding, healing wounds, thrombosis | Pre-reproductive, reproductive, post-reproductive | 12 | 06 | H, M |
| 80. | *Jatropha gossypiifolia* L. | Pinhão-roxo | Childhood allergy to heat, cancer, stroke, general headache, Severe external inflammation, espinhela caída, mouth sore, fogo selvagem, general inflammation, evil eye, miudinho de criança, quebrante, healing wounds | Pre-reproductive, reproductive | 30 | 09 | H, M |
| 81. | *Jatropha mollissima*  (Pohl) Baill. | Pinhão-branco | Toothache | Pre-reproductive | 01 | 01 | H |
| 82. | *Jatropha multifida* L. | Pinhão-de-jardim | Trazer felicidade | Post-reproductive | 01 | 01 | M |
| 83. | *Manihot esculenta* Crantz | Macaxeira | Severe external inflammation | Reproductive | 01 | 01 | M |
| 84. | *Ricinus communis* L. | Mamona | Asthma, general lump on the body, dandruff, constipation, palmar wart, general headache, general body pain, general fever, boil, flu, hemorrhoids, general swelling on the body, urinary tract infection, general inflammation, general external inflammation, inflammation in the breast, blow, spider bite, hair loss, burn, healing wounds, worms, general vomiting | Pre-reproductive, reproductive, post-reproductive | 52 | 25 | H, M |
| 30. | **FABACEAE** |  |  |  |  |  |  |
| 85. | *Amburana cearensis*  (Allemão) A.C.Sm. | Amburana, imburana-de-cheiro | General allergy in the body, chickenpox, ovarian cyst, boil, gastritis, flu, childhood flu, hemorrhage in pregnancy, intestinal infection, urinary infection, inflammation in general, inflammation in the mouth, inflammation in the tongue, inflammation in the uterus, inflammation in the ovaries, cleaning the uterus, dislocation, woman who wants to lose (beginning of spontaneous abortion), childhood measles, healing wounds, healing a child's belly button | Pre-reproductive, reproductive, post-reproductive | 96 | 55 | H, M |
| 86. | *Anadenanthera peregrina*  (L.) Speg. | Angico-preto | Flu, general inflammation, inflammation in the uterus, inflammation in the ovaries | Pre-reproductive, reproductive, post-reproductive | 13 | 10 | H, M |
| 87. | *Bauhinia* sp. | Cipó-de-escada | General back pain | Post-reproductive | 01 | 01 | M |
| 88. | *Cenostigma nordestinum*  Gagnon & G.P.Lewis | Catingueira | Wheezing during childbirth, diarrhea, flu, childhood flu, obesity | Pre-reproductive, reproductive, post-reproductive | 16 | 15 | H, M |
| 89. | *Copaifera* cf.  *langsdorffii* Desf. | Copaíba | Increase immunity, cancer, general body pain, stop bleeding, gastritis, flu, general inflammation, healing wounds, ulcers | Pre-reproductive, reproductive, post-reproductive | 14 | 07 | H, M |
| 90. | *Dipteryx odorata*  (Aubl.) Forsyth f. | Cumaru | General headache, flu, general malaise, pneumonia | Pre-reproductive, reproductive | 05 | 02 | H, M |
| 91. | *Enterolobium*  *contortisiliquum*  (Vell.) Morong | Tamburil | Abortion | Post-reproductive | 01 | 01 | M |
| 92. | *Hymenaea courbaril* L. | Jatobá, jatobá-de-porco | General body pain, flu, urinary tract infection, general inflammation, inflammation of the uterus, inflammation of the ovaries, dislocation, pancada, healing wounds, | Pre-reproductive, reproductive, post-reproductive | 31 | 20 | H, M |
| 93. | *Hymenaea stigonocarpa*  Mart. ex Hayne | Jatobá, jatobá-casca-fina, jataí | Flu | Reproductive, pot-reproductive | 04 | 04 | H, M |
| 94. | *Libidibia ferrea*  (Mart. ex Tul.) L.P.Queiroz | Jucá | General allergy in the body, kidney stones, cancer, general pain in the foot, general pain in the bones, general pain in the kidneys, general fever, chilblains, gastritis, flu, inflammation in general, mycosis, healing wounds, ulcer | Pre-reproductive, reproductive, post-reproductive | 18 | 15 | H, M |
| 95. | *Macropsychanthus*  *grandiflorus*  (Mart. ex Benth.) L.P.Queiroz & Snak | Mucunã | General arm pain | Post-reproductive | 01 | 01 | H |
| 96. | *Mimosa* *caesalpiniifolia* Benth. | Sabiá | Heal a child's belly button, healing wounds | Reproductive, post-reproductive | 02 | 02 | H, M |
| 97. | *Myroxylon peruiferum* L.f. | Bálsamo | Muscle and body pain due to overwork, general back pain, general body pain, general bone pain, fracture, blow | Reproductive, post-reproductive | 10 | 08 | H, M |
| 98. | *Pterocarpus* sp. | Pau-de-sangue | Changes in the menstrual cycle | Post-reproductive | 01 | 01 | H |
| 99. | *Senna alata*  (L.) Roxb. | Canafístula | Flu, hemorrhoids, impinge | Reproductive, post-reproductive | 07 | 06 | H, M |
| 100. | *Senna occidentalis*  (L.) Link | Majerioba, malva | Abortion, changes in the menstrual cycle, flu, inflammation of the uterus, inflammation of the ovaries | Pre-reproductive, post-reproductive | 07 | 06 | H, M |
| 101. | *Tamarindus indica* L. | Tamarinda | Hemorrhoids | Post-reproductive | 01 | 01 | M |
| 102. | *Tephrosia purpurea*  (L.) Pers. | Feijão-de-rolinha | General headache, espinhela caída, Severe external inflammation, wound healing | Pre-reproductive | 04 | 01 | H |
| 103. | *Vachellia farnesiana*  (L.) Wight & Arn. | Coronha, corona | Changes in menstrual cycle, bronchitis, indigestion, stroke, diarrhea, general sore throat, general bone pain, general fever, flu, urinary tract infection, inflammation of the uterus, inflammation of the ovaries | Pre-reproductive, reproductive, post-reproductive | 24 | 18 | H, M |
| 104. | *Vicia faba* L. | Fava-da-Índia | Diarrhea, general fever | Reproductive, post-reproductive | 02 | 02 | H, M |
| 31. | **IRIDACEAE** |  |  |  |  |  |  |
| 105. | *Eleutherine bulbosa*  (Mill.) Urb. | Palmeirinha | General colic, infantile colic, diarrhea, infantile diarrhea, migraine, general infantile fever | Pre-reproductive, reproductive, post-reproductive | 15 | 11 | H, M |
| 32. | **LAMIACEAE** |  |  |  |  |  |  |
| 106. | *Coleus amboinicus* Lour. | Malva-do-reino, malva, malva-cacheada | Flu, general inflammation, inflammation in the uterus, inflammation in the ovaries | Pre-reproductive, reproductive, post-reproductive | 20 | 14 | H, M |
| 107. | *Coleus barbatus* (Andrews) Benth. | Boldo | Changes in the menstrual cycle, heartburn, general colic, indigestion, coronavirus, kidney failure, intestinal discomfort, diarrhea, general stomach ache, general leg pain, general fever, gastritis, liver inflammation, uterus inflammation, ovarian inflammation, general malaise, obesity, hangover | Pre-reproductive, reproductive, post-reproductive | 76 | 49 | H, M |
| 108. | *Coleus comosus* Hochst. ex Gürke | Boldo | Changes in the menstrual cycle, heartburn, general cramps, menstrual cramps, indigestion, coronavirus, kidney deficiency, diarrhea, general stomachache, general headache, general headache, general leg pain, general body pain, general fever, gastritis, flu, liver inflammation, uterus inflammation, ovarian inflammation, general malaise, obesity, hangover, general vomiting | Pre-reproductive, reproductive, post-reproductive | 87 | 54 | H, M |
| 109. | *Lavandula angustifolia* Mill. | Alfazema | General colic, infantile colic, infantile epilepsy, preventing infantile colic, preventing infection in the child's navel | Reproductive, post-reproductive | 12 | 09 | H, M |
| 110. | *Mentha arvensis* L. | Vick, hortelã | Asthma, general colic, infant colic, unblocking the nose, infant headache, general fever, general infant fever, flu, general malaise, sinusitis, general vomiting | Pre-reproductive, reproductive, post-reproductive | 27 | 15 | H, M |
| 111. | *Mentha* ^x^ v*illosa* Huds. | Hortelã | Calm the nerves, kidney stones, general colic, infant colic, diarrhea, infant diarrhea, general headache, general sore throat, earache, general fever, general fever in infants, gas in children, flu, infant flu, general malaise, stuffy nose in children, obesity, prevent headache, general vomiting | Pre-reproductive, reproductive, post-reproductive | 74 | 55 | H, M |
| 112. | *Mesosphaerum*  *suaveolens*  (L.) Kuntze | Bamburral | Indigestion | Post-reproductive | 01 | 01 | H |
| 113. | *Ocimum basilicum* L. | Majericão | Earache, flu, cold | Post-reproductive | 04 | 03 | H, M |
| 114. | *Ocimum gratissimum* L. | Chá-pra-tudo | General allergy in the body, chicken pox, menstrual cramps, indigestion, general pain in the body, general fever, gas buildup, flu, intestinal infection | Pre-reproductive, reproductive, post-reproductive | 13 | 10 | H, M |
| 115. | *Rosmarinus*  *officinalis* L. | Alecrim | Calm nerves, infant colic, menstrual colics, earache, general body pain, flu | Pre-reproductive, reproductive, post-reproductive | 06 | 04 | H, M |
| 33. | **LAURACEAE** |  |  |  |  |  |  |
| 116. | *Cinnamomum verum*  J.Presl | Canela | Changes in menstrual cycle, menstrual colics, general body aches, general dizziness | Pre-reproductive, reproductive | 04 | 03 | H, M |
| 117. | *Persea americana* Mill. | Abacate | Kidney stones, cancer, dandruff, hair loss | Reproductive, post-reproductive | 07 | 06 | H, M |
| 34. | **LECYTHIDACEAE** |  |  |  |  |  |  |
| 118. | *Bertholletia excelsa* Bonpl. | Castanha-do-Pará | Insect bite | Reproductive | 01 | 01 | M |
| 35 | **LYTHRACEAE** |  |  |  |  |  |  |
| 119. | *Punica granatum* L. | Romã | General sore throat, gastritis, flu, general inflammation of the throat. Inflammation of the tonsils, pneumonia, prevent cancer, reflux | Pre-reproductive, reproductive, post-reproductive | 15 | 10 | H, M |
| 36. | **MALPIGHIACEAE** |  |  |  |  |  |  |
| 120. | *Malpighia emarginata* DC. | Acerola | Flu | Reproductive, post-reproductive | 04 | 04 | H, M |
| 37. | **MALVACEAE** |  |  |  |  |  |  |
| 121. | *Abelmoschus esculentus*  (L.) Moench | Quiabo | Acne | Pre-reproductive | 01 | 01 | H |
| 122. | *Gossypium herbaceum* L. | Algodão, algodão-roxo | Growing heart, furuncle, stroke, Severe external inflammation, inflammation of the uterus, inflammation of the ovaries, quebrante | Pre-reproductive, reproductive, post-reproductive | 09 | 06 | H, M |
| 123. | *Gossypium hirsutum* L. | Algodão, algodão-branco | Stroke, furuncle, flu, intestinal infection, inflammation of the uterus, inflammation of the ovaries, general vomiting | Pre-reproductive, reproductive, post-reproductive | 07 | 06 | H, M |
| 124. | *Hibiscus* sp. | Hibisco | Obesity | Post-reproductive | 01 | 01 | M |
| 125. | *Luehea candicans* Mart. | Açoita-cavalo | Changes in the menstrual cycle, blood cramps, general colic, diarrhea, gastritis, flu, urinary tract infection, inflammation of the uterus, inflammation of the ovaries, inflammation of the kidneys | Reproductive, post-reproductive | 15 | 13 | H, M |
| 126. | *Pavonia cancellata*  (L.) Cav. | Barba-de-veado | Inflammation in the uterus, inflammation in the ovaries | Post-reproductive | 01 | 01 | M |
| 38. | **MARANTACEAE** |  |  |  |  |  |  |
| 127. | *Maranta arundinacea* L. | Araruta | Hemorrhoids, worms | Post-reproductive | 02 | 02 | H, M |
| 39. | **MELIACEAE** |  |  |  |  |  |  |
| 128. | *Carapa guianensis* Aubl. | Andiroba, indiroba | Gastritis, flu, general inflammation of the throat, healing wounds | Pre-reproductive, reproductive, post-reproductive | 08 | 04 | H, M |
| 40. | **MORACEAE** |  |  |  |  |  |  |
| 129. | *Ficus* sp. | Figueira | Indigestion | Reproductive | 02 | 01 | H |
| 130. | *Maclura tinctoria*  (L.) D.Don ex Steud. | Janaguba | General body pain, gastritis, dislocation | Pre-reproductive, post-reproductive | 04 | 03 | H, M |
| 41. | **MUSACEAE** |  |  |  |  |  |  |
| 131. | *Musa* ^x^ *paradisiaca* L. | Bananeira | Depression, flu | Pre-reproductive | 02 | 02 | H, M |
| 42. | **MYRTACEAE** |  |  |  |  |  |  |
| 132. | *Campomanesia aromatica*  (Aubl.) Griseb. | Guabiraba | Chickenpox | Post-reproductive | 01 | 01 | M |
| 133. | *Eucalyptus globulus*  Labill. | Eucalipto | General fever, flu, general inflammation | Pre-reproductive, reproductive, post-reproductive | 17 | 15 | H, M |
| 134. | *Psidium guajava* L. | Goiaba, goiaba-comum | Heartburn, blood cramp, cancer, indigestion, diarrhea, childhood diarrhea, general stomach pain, general fever, flu, intestinal infection, inflammation of the uterus, inflammation of the ovaries, hair loss, general vomiting | Pre-reproductive, reproductive, post-reproductive | 31 | 24 | H, M |
| 135. | *Syzygium*  *aromaticum*  (L.) Merr. & L.M.Perry | Cravim | Calm the nerves, arrhythmia, constipation, general headache, general body pain, flu, general heart problem | Pre-reproductive, reproductive, post-reproductive | 13 | 10 | H, M |
| 136. | *Syzygium* sp. | Azeitona | Diabetes | Reproductive | 01 | 01 | M |
| 43. | **NYCTAGINACEAE** |  |  |  |  |  |  |
| 137. | *Boerhavia*  *diffusa L.* | Pega-pinto | Asthma, urinary tract infection, inflammation of the uterus, inflammation of the ovaries, general dizziness | Pre-reproductive, post-reproductive | 05 | 04 | H, M |
| 44. | **NYMPHAECEAE** |  |  |  |  |  |  |
| 138. | *Nymphaea amazonum*  Mart. & Zucc. | Vitória-régia | He only knows that the resource is medicine | Pre-reproductive reproductive | 02 | 02 | H |
| 45. | **PASSIFLORACEAE** |  |  |  |  |  |  |
| 139. | *Passiflora edulis* Sims | Maracujá | Calm the nerves, general weakness in the body, general malaise | Pre-reproductive, post-reproductive | 03 | 02 | M |
| 46. | **PEDALIACEAE** |  |  |  |  |  |  |
| 140. | *Sesamum indicum* L. | Gergelim, gergelim-preto, gergelim-branco, gergelim-amarelo | Bronchitis, general lump on the body, palmar wart, stroke, diarrhea, general headache, general sore throat, earache, labor pain, general leg pain, general fever, furuncle, flu, general swelling on the body, urinary tract infection, general inflammation, inflammation in the breast, blow, thyroid problems, hair loss, healing wounds, stye, general vomiting | Pre-reproductive, reproductive, post-reproductive | 62 | 31 | H, M |
| 47. | **PHYLLANTHACEAE** |  |  |  |  |  |  |
| 141. | *Phyllanthus niruri* L. | Quebra-pedra | Gallstones, kidney stones, general colic, general back pain, general kidney pain, general fever, urinary tract infection, prostate inflammation | Pre-reproductive, reproductive, post-reproductive | 24 | 21 | H, M |
| 48. | **PHYTOLACCACEAE** |  |  |  |  |  |  |
| 142. | *Petiveria alliacea* L. | Tipi | General body pain, general bone pain, flu, evil eye, snake bite, cold, rheumatism | Pre-reproductive, reproductive | 10 | 08 | H, M |
| 49. | **PIPERACEAE** |  |  |  |  |  |  |
| 143. | *Piper tuberculatum* Jacq. | Pimenta-de-macaco | General body pain | Post-reproductive | 01 | 01 | M |
| 50. | **PLANTAGINACEAE** |  |  |  |  |  |  |
| 144. | *Scoparia dulcis* L. | Vassourinha | Child allergy to heat, general colic, urinary infection, general inflammation, inflammation in the uterus, inflammation in the ovaries, miudinho de criança, general dizziness | Pre-reproductive, reproductive, post-reproductive | 16 | 14 | H, M |
| 51. | **POACEAE** |  |  |  |  |  |  |
| 145. | *Cymbopogon citratus*  (DC.) Stapf | Capim-santo, capim-limão | Calm the nerves, general colic, infant colic, menstrual colic, indigestion, diarrhea, general stomachache, general headache, general body pain, migraine, general fever, general weakness in the body, flu, high blood pressure, low blood pressure, intestinal infection, general inflammation, general malaise | Pre-reproductive, reproductive, post-reproductive | 74 | 56 | H, M |
| 52. | **RHAMNACEAE** |  |  |  |  |  |  |
| 146. | *Ampelozizyphus* cf. *amazonicus* Ducke | Cervejinha-preta | Malaria | Reproductive | 01 | 01 | H |
| 147. | *Sarcomphalus*  *joazeiro*  (Mart.) Hauenschild | Juá, juazeiro | Tongue inflammation | Pre-reproductive, reproductive | 02 | 02 | H, M |
| 53. | **ROSACEAE** |  |  |  |  |  |  |
| 148. | *Malus domestica* (Suckow) Borkh. | Maçã | Healing wounds | Post-reproductive | 01 | 01 | M |
| 54. | **RUBIACEAE** |  |  |  |  |  |  |
| 149. | *Coffea arabica* L. | Café | Worms | Post-reproductive | 01 | 01 | M |
| 150. | *Genipa americana* L. | Jenipapo | Dislocation | Pre-reproductive | 02 | 02 | H |
| 151. | *Morinda citrifolia* L. | Noni | Scratching in the uterus | Pre-reproductive, reproductive | 02 | 02 | M |
| 152. | *Tocoyena formosa*  (Cham. & Schltdl.) K.Schum. | Jenipapo, Jeniparana | Fracture, dislocation, wound healing | Pre-reproductive, reproductive, post-reproductive | 26 | 23 | H, M |
| 55. | **RUTACEAE** |  |  |  |  |  |  |
| 153. | *Citrus sinensis*  (L.) Osbeck | Laranja | Calm the nerves, anemia, heartburn, infant colic, indigestion, diarrhea, general stomachache, general headache, general fever, flu, high blood pressure, low blood pressure, intestinal infection, general malaise, obesity, general vomiting | Pre-reproductive, reproductive, post-reproductive | 44 | 34 | H, M |
| 154. | *Citrus* ^x^ *limon* (L.) Osbeck | Limão | Cholesterol, general colic, indigestion, coronavirus, diarrhea, general stomachache, general headache, general fever, flu, obesity, general vomiting | Pre-reproductive, reproductive, post-reproductive | 24 | 16 | H, M |
| 155. | *Ruta graveolens* L. | Arruda | Abortion, general colic, infant colic, menstrual colic, coronavirus, general headache, earache, gas buildup, eye infection, urinary infection, general inflammation, general malaise, evil eye, snake bite | Pre-reproductive, reproductive, post-reproductive | 30 | 17 | H, M |
| 56. | **SAPOTACEAE** |  |  |  |  |  |  |
| 156. | *Pouteria ramiflora*  (Mart.) Radlk. | Pitomba-de-leite | Gastritis, hemorrhoids | Pre-reproductive, post-reproductive | 03 | 02 | H |
| 57. | **SMILACEAE** |  |  |  |  |  |  |
| 157. | *Smilax* sp. | Japecanga | Flu, inflammation of the uterus, inflammation of the ovaries | Post-reproductive | 05 | 02 | H, M |
| 58. | **SOLANACEAE** |  |  |  |  |  |  |
| 158. | *Nicotiana tabacum* L. | Fumo | Dislocation | Pre-reproductive | 02 | 02 | H |
| 159. | *Solanum agrarium*  Sendtn. | Melancia-da-praia | Changes in the menstrual cycle | Post-reproductive | 01 | 01 | M |
| 160. | *Solanum crinitum*  Lam. | Jurubeba | Furuncle | Reproductive | 01 | 01 | H |
| 161. | *Solanum fernandesii*  V.S. Samp. & R. Moura | Melancia-da-praia | Changes in the menstrual cycle | Post-reproductive | 01 | 01 | M |
| 162. | *Solanum melongena* L. | Beringela | Diabetes | Pre-reproductive, reproductive | 02 | 02 | H |
| 163. | *Solanum tuberosum* L. | Batata-inglesa | Gastritis | Reproductive | 01 | 01 | M |
| 59. | **TURNERACEAE** |  |  |  |  |  |  |
| 164. | *Turnera* cf. *coerulea* DC. | Xanana | Urinary tract infection, furuncle | Pre-reproductive | 01 | 01 | H |
| 165. | *Turnera pumilea* L. | Xanana | Urinary tract infection, furuncle | Pre-reproductive | 01 | 01 | H |
| 166. | *Turnera scabra* Millsp. | Xanana | Urinary tract infection, furuncle, general dizziness | Pre-reproductive | 04 | 03 | H |
| 167. | *Turnera* sp. | Xanana | Urinary tract infection, furuncle | Pre-reproductive | 01 | 01 | H |
| 168. | *Turnera subulata* Sm. | Xanana | Urinary tract infection, furuncle, general dizziness | Pre-reproductive | 04 | 03 | H |
| 60. | **URTICACEAE** |  |  |  |  |  |  |
| 169. | *Cecropia glaziovii*  Snethl. | Torém | Kidney stones, urinary tract infection, gastritis | Pre-reproductive, post-reproductive | 04 | 03 | H, M |
| 61 | **VERBENACEAE** |  |  |  |  |  |  |
| 170. | *Lippia alba*  (Mill.) N.E.Br. ex Britton & P.Wilson | Cidreira, erva-cidreira | Calm the nerves, heartburn, infant colic, indigestion, diarrhea, general stomach ache, general body pain, general fever, gas, gastritis, flu, high blood pressure, low blood pressure, stomach infection, intestinal infection, general malaise, general dizziness, general vomiting | Pre-reproductive, reproductive, post-reproductive | 96 | 67 | H, M |
| 171. | *Lippia origanoides* Kunth | Cidreira-do-mato | Gripe, infecção urinária | Pre-reproductive | 02 | 01 | H |
| 62. | **VITACEAE** |  |  |  |  |  |  |
| 172. | *Cissus verticillata*  (L.) Nicolson & C.E.Jarvis | Tiú | He only knows that the resource is medicine | Post-reproductive | 01 | 01 | H |
| 63. | **VOCHYSIACEAE** |  |  |  |  |  |  |
| 173. | *Qualea parviflora* Mart. | Pau-terra | General bone pain, gastritis | Reproductive, post-reproductive | 04 | 03 | H, M |
| 64. | **XIMENIACEAE** |  |  |  |  |  |  |
| 174. | *Ximenia americana* L. | Ameixa | Changes in the menstrual cycle, anemia, cancer, menstrual cramps, general headache, general body pain, general bone pain, Severe external inflammation, gastritis, flu, childhood flu, urinary tract infection, general inflammation, tooth inflammation, inflammation of the uterus, inflammation of the ovaries, inflammation of the kidneys, dislocation, general malaise, obesity, healing wounds, healing a child's belly button | Pre-reproductive, reproductive, post-reproductive | 156 | 78 | H, M |
| 65. | **ZINGIBERACEAE** |  |  |  |  |  |  |
| 175. | *Curcuma longa* L. | Açafroa, gengibre-amarelo, gengibre | Coronavirus, general sore throat, general body pain, migraine, general fever, flu, intestinal infection, urinary infection, general inflammation of the throat, inflammation in the uterus, inflammation in the ovaries, obesity | Pre-reproductive, reproductive, post-reproductive | 34 | 24 | H, M |
| 176. | *Zingiber officinale*  Roscoe | Gengibre, gengibre-branco | Diarrhea, general headache, general body pain, migraine, general fever, flu, intestinal infection, urinary infection, general inflammation of the throat, inflammation of the uterus, inflammation of the ovaries, obesity, general vomiting | Pre-reproductive, reproductive, post-reproductive | 27 | 14 | H, M |
|  | **NOT IDENTIFIED (NI)** |  |  |  |  |  |  |
| 177. | NI1 | Cansanção-branco | Cancer | Post-reproductive | 02 | 02 | M |
| 178. | NI2 | Dipirona | General body pain | Post-reproductive | 02 | 02 | M |
| 179. | NI3 | Enxofre | General body pain, inflammation of the uterus, inflammation of the ovaries | Post-reproductive | 03 | 02 | H, M |
| 180. | NI4 | Novalgina | General body aches, general fever | Post-reproductive | 02 | 02 | M |
| 181. | NI5 | Poêjio | General inflammation of the throat | Reproductive | 01 | 01 | M |

**Table 2** Descriptions of the cases and conflicting points of the five participants whose data were excluded from the analyses

| **Cases** | **Conflicting point** |
| --- | --- |
| #1 Male individual, 31 years old, has already started a family, is currently divorced, has no children, lives with his parents, on whom he depends financially | He presents characteristics of the pre-reproductive (not married and depends on his parents for resources – incorporated capital), and reproductive (he has had the experience of forming a family) stages. By including the individual in the pre-reproductive stage, we would have an incorrect analysis of the data, as the participant has already formed a family and we are considering that these experiences increase knowledge about plants. Including it in the reproductive stage would be another weak point, as the individual does not have children, is not married or in a stable union, and depends heavily on his parents for his support. |
| # 2 Similar case to the previous one described. Female individual, 24 years old, has already started a family, is currently divorced, has no children, lives with her parents, on whom she depends financially | Same conflicting point as the previous one |
| # 3 Similar case to the one described previously. Female individual, 29 years old, has already started a family, is currently divorced, has no children, lives with her parents, on whom she depends financially | Same conflicting point as the previous one |
| # 4 Male individual, 41 years old, has never started a family and has no children, currently lives with an aunt, on whom he is not financially dependent. He has no expectations of having children | The individual presents many conflicting points and including at any stage we could commit false positives. The individual is self-sufficient and does not depend on adults to support himself, not allowing him to be included in the pre-reproductive stage, even if he has not had the experience of forming a family or having children, nor does he have characteristics to include him in the reproductive stage, as He is not starting a family and has no children. The individual has no expectations of having children, which could well be a target to place them in the post-reproductive stage, even so we would make mistakes when doing so, as we consider the experiences lived in the reproductive and post-reproductive stages (in which the offspring has demands for resources) are important for the development and differentiation of knowledge about plants. The individual has not experienced these experiences |
| # 5 Female individual, 33 years old, has already started a family and has two male children, currently lives with her parents, on whom she is financially dependent. She did not go into menopause. No expectations of having children | She presents some conflicting points, which include: she depends heavily on parents' resources for her and her children's support; is not currently forming a family and; even though she has not entered menopause, she has no expectations of having children, which could lead to mistakes in classifying her at any stage of life due to her complex experiences |
